# *Sarcocystis* infection in red deer *(Cervus elaphus)* with eosinophilic myositis/fasciitis in Switzerland and involvement of red foxes (*Vulpesvulpes*) and hunting dogs in the transmission

**DOI:** 10.1016/j.ijppaw.2020.09.005

**Published:** 2020-10-01

**Authors:** Walter Basso, Cristian A. Alvarez Rojas, Daniel Buob, Maja Ruetten, Peter Deplazes

**Affiliations:** a Institute of Parasitology, Vetsuisse-Faculty, University of Zurich, Winterthurerstrasse 266a, CH, 8057, Zurich, Switzerland; b Institute of Parasitology, Vetsuisse-Faculty, University of Bern, Länggassstrasse 122, CH, 3012, Bern, Switzerland; c Institute of Veterinary Pathology, University of Zurich, Winterthurerstrasse 286, CH, 8057, Switzerland

**Keywords:** *Sarcocystis*, Red deer (*Cervus elaphus*), Red fox (*Vulpes vulpes*), Dog (*Canis familiaris*), Myositis/fasciitis, Molecular identification

## Abstract

Red deer (*Cervus elaphus*) carcasses showing grey-greenish discolouration have been increasingly observed in the canton of Grisons, Switzerland. We investigated whether *Sarcocystis* infections were associated with this pathology, and whether wild and domestic canids were involved in their transmission. Meat from affected red deer (*n =* 26), faeces and intestines from red foxes (*Vulpesvulpes*) (*n =* 126), and faeces from hunting dogs (*n =* 12) from the region, were analysed. Eosinophilic myositis and/or fasciitis were diagnosed in 69% of the deer, and sarcocysts were observed in 89% of the animals. Molecular typing targeting a ~700bp variable region of the 18S rRNA gene revealed *Sarcocystis hjorti* in 73%, *S. venatoria/S. iberica* in 54%, *S. linearis/S. taeniata* in 12%, *S. pilosa* in 8% and *S. ovalis* in 4% of the deer samples. No inflammatory changes were observed in red deer carcasses with normal appearance (*n* = 8); however, sarcocysts were observed in one sample, and *S. hjorti*, *S. venatoria/S. iberica* or *S. silva* DNA was detected in five samples. *Sarcocystis* oocysts/sporocysts were observed in 11/106 faecal and 6/20 intestinal fox samples, and in 2/12 canine samples. *Sarcocystis tenella* (*n =* 8), *S. hjorti* (*n =* 2), *S. gracilis* (*n =* 2), and *S. miescheriana* (*n =* 1) were identified in foxes, and *S. gracilis* (*n* = 2), *S. capreolicanis* (*n* = 1) and *S. linearis/S. taeniata* (*n* = 1) in dogs. This study provides first molecular evidence of *S. pilosa* and *S. silva* infection in red deer and *S. linearis/S. taeniata* in dogs and represents the first record of *S. ovalis* transmitted by corvids in Central Europe. Although *Sarcocystis* species infecting red deer are not regarded as zoonotic, the affected carcasses can be declared as unfit for human consumption due to the extensive pathological changes.

## Highlights

• Red deer with eosinophilic myositis/fasciitis were infected with *Sarcocystis*.
• Red foxes and hunting dogs in Grisons transmit *Sarcocystis* spp. to cervids, ruminants and swine.
• *Sarcocystis ovalis*, transmitted by corvid birds, infect red deer in Central Europe.
• *Sarcocystis silva* and *S. pilosa* were first detected in red deer by molecular methods.
• Red foxes are natural definitive hosts of *Sarcocystis hjorti.*

## Graphical abstract

**Figure:**
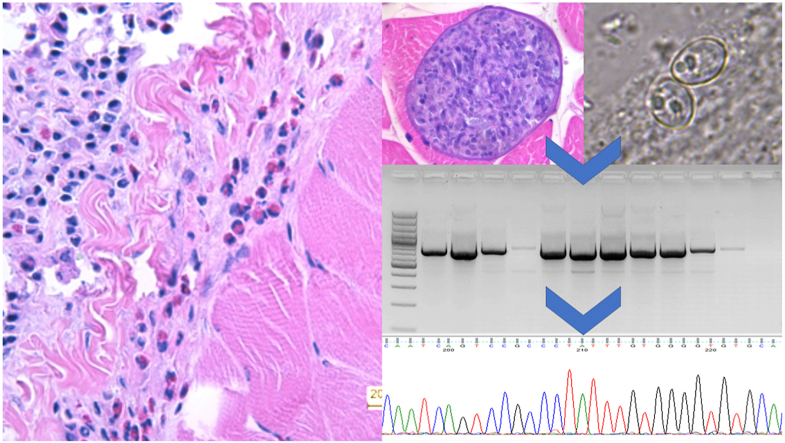

## 1 Introduction

Members of the genus *Sarcocystis* (Apicomplexa, Sarcocystidae) are heteroxenous parasites with carnivores as definitive hosts (DH) and herbivores as intermediate hosts (IH) (Dubey et al., 2016; Deplazes et al., 2016). *Sarcocystis* spp. undergo a sexual reproduction in the intestine of the DH leading to production of sporulated oocysts, which are shed to the environment with the faeces, and serve as source of infection for IH. Definitive hosts may shed oocysts/sporocysts over several months, being responsible for prolonged environmental contamination (Dubey et al., 2016; Deplazes et al., 2016). Intermediate hosts acquire the infection by ingestion of sporocysts contaminating food or water. In the IH, the parasite undergoes an asexual merogonic reproduction infecting endothelial cells of several organs and later, muscle or nerve cells, in which tissue cysts (“sarcocysts”) containing numerous zoites (infectious for DH) are built (Dubey et al., 2016; Deplazes et al., 2016).

More than 200 species of *Sarcocystis* with variable pathogenicity have been described infecting mammals (including humans), reptiles and birds worldwide (Dubey et al., 2016). Intestinal infections in the DH are generally asymptomatic, except in humans (Fayer et al., 2015). In the IH the course of infection is frequently subclinical, but it may be severe and also fatal depending on the *Sarcocystis* species. Clinical signs such as fever, weakness, cyanosis, dyspnoea, neurological signs, abortion and death have been described in several animal species after experimental (Johnson et al., 1975; Koller et al., 1977) and natural (Caspari et al., 2011; Ravi et al., 2015) infections.

In cervids, clinical *Sarcocystis* infections are considered rare; however, some species were shown to be pathogenic in experimental infections. Mule deer (*Odocoileus hemionus*) fawns inoculated with 50,000 to 1 million sporocysts of *S. hemionilatrantis* became anorectic, showed incoordination, and died between 27 and 63 days post-inoculation (Koller et al., 1977). Rocky mountain elk (*Cervus elaphus*), inoculated with 250,000 sporocysts of *Sarcocystis* spp. (including *S. sybillensis* and *S. wapiti*) showed reduced weight gain associated with higher parasite burdens in different tissues, when compared with non-inoculated control animals (Foreyt et al., 1995). Furthermore, a natural case of clinical acute infection with *S. alceslatrans* was recently described in a moose (*Alces*) calf presenting neurological signs and multisystemic inflammation with presence of intralesional schizonts in the brain, the uveal tract of both eyes, and also in lungs, heart and kidneys (Ravi et al., 2015).

In September 2010 and October 2011, two muscle samples of hunted red deer (*Cervus elaphus*) from Grisons, Switzerland, showing a grey-greenish discolouration and a gelatinous change of the fasciae were sent to the Institute for Food Safety and Hygiene, Vetsuisse Faculty, University of Zurich, Switzerland for analysis. Histopathologically, eosinophilic fasciitis was observed and *S. hjorti* was assumed as the causing agent of this pathology (Stephan et al., 2012). This finding was uncommon, but according to the Hunting and Fishing Department of Grisons and to regional meat inspectors, hunted red deer with greenish tissue discolouration have been increasingly observed over the last few years, as it was also informed by the Journal for Swiss hunters (Deutz, 2013). Even though the *Sarcocystis* species found in red deer are not regarded as zoonotic, the affected carcasses can be declared as unfit for human consumption due to the extensive pathological changes (Stephan et al., 2012). Association of *Sarcocystis* infection with greenish discolouration of the carcasses due to eosinophilic myositis/fasciitis has been described in several animal species including cattle, sheep, horses and South American camelids (Gajadhar et al., 1987; Wouda et al., 2006; Aráoz et al., 2019; Jensen et al., 1986; Vangeel et al., 2013; Herd et al., 2015; La Perle et al., 1999), but this was considered infrequent in cervids (Stephan et al., 2012).

Although the occurrence of *Sarcocystis* infections in cervids has been known for a long time, most *Sarcocystis* species have been only recently described, and many aspects about their epidemiology and significance remain still unknown (Dubey et al., 2016). At least eleven *Sarcocystis* species have been detected in European red deer: i.e. *S. hjorti, S. hardangeri, S. ovalis, S. tarandi, S. cervicanis, S. truncata, S. elongata, S. linearis, S. iberica, S. venatoria* and *S. morae* (Dahlgren and Gjerde, 2010a; Gjerde, 2014b; Hernández-Rodriguez et al., 1981; Gjerde et al., 2017b; Dubey et al., 2016), but the DH for only a few of these species have been identified so far (Dubey et al., 2016; Gjerde et al., 2017b; Irie et al., 2017; Dahlgren and Gjerde, 2010b).

This study aimed to identify the *Sarcocystis* species infecting red deer with eosinophilic myositis/fasciitis, and with normal carcass appearance in Switzerland, and to investigate the possible involvement of red foxes (*Vulpes* *vulpes*) and hunting dogs as definitive hosts of these species.

## 2 Materials and methods

### 2.1 Red deer samples

Meat samples from 26 red deer showing grey-greenish discolouration and gelatinous changes in fasciae and muscles (Fig. 1)(age range = 1–13 years, mean 2.0 years; females *n* = 18, males *n* = 8) were collected for diagnosis of *Sarcocystis* infection by meat inspectors in four regions of the canton of Grisons, Switzerland (Davos [Deer 1–6]; Rueun [Deer 7–9]; Cunters [Deer 10–20] and Filisur [Deer 21–26]) during the hunting season 2015 (June to September). Additionally, samples from eight red deer with normal carcass appearance (Deer 27–34) (age range 2–3 years, mean = 2.5 years; females *n* = 2, males *n* = 6) hunted in the same region (i.e. Rueun) during the same hunting season were included as a control group. Estimated age and sex of the sampled animals, as well as the intensity of the observed macroscopical changes, are indicated in Table 1. Geographical coordinates of the hunting sites and sampling dates are registered in Supplementary Table 1. From each killed animal (*n* = 34), a sample (~10 × 15 × 5 cm) of the limb muscles was collected. In some cases (*n* = 11), samples from diaphragm showing grey-greenish discolouration were additionally included (Table 1). All the samples were immediately refrigerated and sent to the Institute of Parasitology, University of Zurich for further examination.

**Fig. 1:**
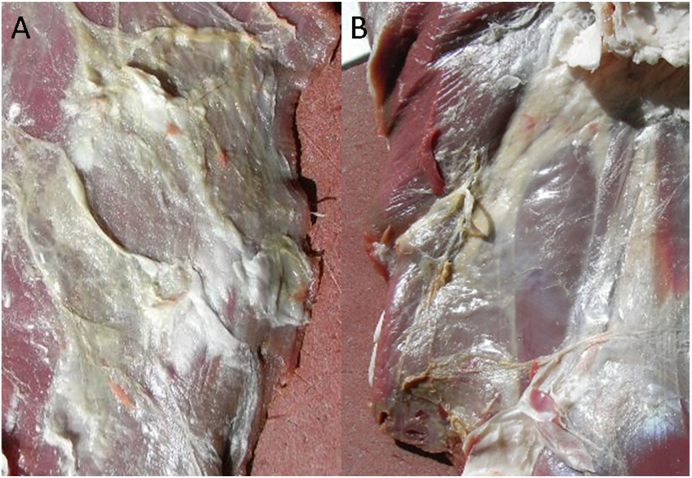
A–B: Grey-greenish discolouration of muscle and fasciae from red deer. (Photo: E. Eggenberger) (For interpretation of the references to colour in this figure legend, the reader is referred to the Web version of this article.)

**Table 1:** Red deer from the Canton of Grisons, Switzerland, analysed for *Sarcocystis* infection by histopathological and molecular techniques.

Hunting region	Deer No	Age (years)	Sex (F/M)	Macroscopical changes in the carcass^a)^	Histology in skeletal muscle samples^b)^			*Sarcocystis* PCR (Pos/Neg)	*Sarcocystis* species identification (Y/N/n)	
					Eosinophilic-lymphoplasmacellular fasciitis (L/D)	Eosinophilic myositis (L/D)	Presence of sarcocysts (L/D)		By direct sequencing	By cloning
Davos	1	2	M	++	+++/+++	+/+	Y/Y	Pos	N	Y
	2	2	M	+++	+/++	+/+	Y/Y	Pos	N	Y
	3	1	F	++	+/+	+/+	Y/Y	Pos	N	Y
	4	1	F	++	+/+	+/−	N/N	Pos	Y	Y
	5	2	M	+	+/−	+/−	Y/Y	Pos	Y	Y
	6	2	F	+++	+++/+	+/+	Y/Y	Pos	Y	Y
Rueun	7	1	F	+++	+++/+++	+/+	Y/Y	Pos	Y	Y
	8	4–6	F	+++	+++/n	+/n	N/n	Pos	N	Y
	9	1	F	+++	++/n	+/n	Y/n	Pos	N	Y
Cunters	10	1	F	+	–/n	–/n	Y/n	Pos	N	Y
	11	1	M	++	+/n	+/n	Y/n	Pos	N	Y
	12	2	M	+	–/n	–/n	N/n	Pos	N	Y
	13	2	F	+	–/n	–/n	Y/n	Pos	N	Y
	14	1	F	++	–/n	–/n	Y/n	Pos	Y	Y
	15	1	F	+	++/n	–/n	Y/n	Pos	N	Y
	16	1	F	++	–/n	+/n	Y/n	Pos	Y	n
	17	3	M	++	+/n	+/n	Y/n	Pos	N	Y
	18	1	F	+	–/n	+/n	Y/n	Pos	N	Y
	19	2	M	++	–/n	–/n	Y/n	Pos	N	Y
	20	1	F	+	–/n	–/n	Y/n	Pos	Y	Y
Filisur	21	1	F	+	+/+	+/+	Y/N	Pos	Y	Y
	22	1	F	+++	+/−	+/−	Y/Y	Pos	N	Y
	23	1	F	+++	+/+	+/+	Y/Y	Pos	N	Y
	24	1	F	+	+/+	+/+	Y/N	Pos	N	Y
	25	1	F	++	+/−	+/−	Y/Y	Pos	N	Y
	26	13	M	+	−/−	−/−	Y/N	Pos	N	Y
Rueun	27	2	M	–	–/n	–/n	N/n	Neg	n	n
	28	3	F	–	–/n	–/n	N/n	Neg	n	n
	29	2	M	–	–/n	–/n	N/n	Pos	N	Y
	30	2	F	–	–/n	–/n	N/n	Pos	N	Y
	31	3	M	–	–/n	–/n	N/n	Neg	n	n
	32	3	M	–	–/n	–/n	Y/n	Pos	N	Y
	33	3	M	–	–/n	–/n	N/n	Pos	N	Y
	34	2	M	–	–/n	–/n	N/n	Pos	N	Y

F: female; M: male; a) macroscopical changes: –: normal carcass appearance, +: slight, ++: moderate, +++: marked grey-greenish discolouration areas in the carcass.

b) microscopical changes: –: no pathological changes, +: slight, ++: moderate, +++: severe fasciitis or myositis, Y: yes, N: no; n: not examined; L: limb muscle; D: diaphragm; Pos: positive PCR result for *Sarcocystis* DNA; Neg: negative PCR result for *Sarcocystis* DNA.

### 2.2 Red fox samples

Faecal samples (*n* = 106; Fox 1–106) and intestines (*n* = 20; Fox 107–126) from red foxes were collected by hunters all over Grisons for two years (2013–2015). The geographic coordinates of the collection or hunting sites and age and sex of the hunted foxes were registered in Supplementary Table 2. All samples were frozen at −80 °C for at least two weeks to inactivate potentially present *Echinococcus multilocularis* eggs and were subsequently analysed for *Sarcocystis* infection. The foxes were hunted for reasons independent of this study and the hunters submitted the examined material on a voluntary basis.

### 2.3 Dog samples

In addition, in winter 2015, faecal samples from hunting dogs from Grisons (*n* = 12: Dog 1–12) were collected and analysed for *Sarcocystis* infection. The samples were directly submitted by the hunters to the Institute of Parasitology together with a questionnaire including data from the dogs (i.e. breed, age, sex, contact with deer, feeding habits and the possibility of eating raw meat or viscera from red deer and other hunted animals). The faecal samples were processed as indicated above for fox samples. Collected data from the dogs are displayed in Supplementary Table 3.

### 2.4 Histopathology

About 40 g of fresh meat from each red deer were fixed in 4% buffered formalin on the day of receiving the samples. Within 24 h, formalin-fixed tissues were embedded in paraffin, sectioned at 2.0–3.0 μm, and stained with haematoxylin and eosin (HE) for routine histopathological examination. All collected meat samples were evaluated for the presence of *Sarcocystis* and histological abnormalities.

### 2.5 Isolation of *Sarcocystis* oocysts/sporocysts from faeces and intestine

Faecal samples from foxes and dogs were processed for coproscopy by a flotation method using a concentrated sucrose solution (specific gravity of 1.3 g/l). After centrifugation at 500*g* for 5 min, three drops from the surface of the flotation fluid were examined at 100× and 400× magnification using a Leica DM 1000 LED microscope. In positive samples, the rest of the supernatant was collected and processed for isolation of *Sarcocystis* oocysts/sporocysts as previously described (Schares, 2007). Small intestines from red foxes were cut longitudinally and scrapings from the mucosa were taken using a disposable scalpel blade. The scrapings were homogenised and processed by flotation as indicated for faecal samples above.

### 2.6 DNA extraction

DNA was extracted from meat samples previously frozen at −20 °C (500 mg/animal including either limb muscles or limb muscles and diaphragm if this sample was additionally collected) using the QIAamp DNA mini kit (QIAGEN, Hilden, Germany) as described by (Glor et al., 2013). Besides, DNA was extracted from *Sarcocystis* oocysts/sporocysts isolated from fox or dog faeces concentrated in 200 μl aqueous solution using the ZR Fecal DNA MiniPrep kit (Zymo Research, USA), as indicated by the manufacturer.

### 2.7 Polymerase chain reaction (PCR) and sequencing

DNA samples obtained by the different extraction methods explained above were tested by a PCR targeting a variable ~700 bp region of the 18S rRNA gene of *Sarcocystis* using the primers SarcoF/SarcoR (Moré et al., 2011). The amplification reactions (initial denaturation step at 94 °C for 15 min followed by 40 cycles of 94 °C 40 s; 59 °C 30s; 72 °C 1 min and a final extension step at 72 °C for 10 min) were performed in a thermocycler (SensoQuest Labcycler) in a final volume of 50 μl, using 25 μl QIAGEN Multiplex Mastermix; 19 μl QIAGEN RNase-free water; 0.5 μl of each primer (100 μM solution) and 5 μl DNA/sample. The amplification products were analysed by gel electrophoresis in 1.5% agarose stained with GelRed (Biotium). PCR products exhibiting the expected fragment length were purified using the MinElute PCR Purification kit (QIAGEN, Hilden, Germany) and further sequenced in both directions with the same primers used for PCR (Synergene Biotech GmbH, Schlieren and Microsynth, Switzerland) to assess the *Sarcocystis* species involved in the infections. Additionally, amplified PCR products were further cloned using the TOPO™ TA Cloning™ Kit (Thermo Scientific). Five clones per sample were selected and sequenced using internal vector primers. The obtained *Sarcocystis* spp. sequences from red deer, red foxes and dogs were submitted to GenBank® (accession numbers listed in Table 2) and compared with available sequences in GenBank® using the megablast function of BLASTn (http://blast.ncbi.nlm.nih.gov).

**Table 2:** Description of *Sarcocystis* spp. 18S rRNA gene sequences amplified from skeletal muscle of red deer from Grisons, Switzerland, obtained by direct sequencing of the PCR products and after cloning into vector plasmids.

Animal ID	Sequence ID	Sequence length (bp)	BLASTn identity	(%)	GenBank® accession no. (reference sequences)	Reference	*Sarcocystis* sp. (this study)	GenBank® accession no. (this study)
Deer 1	D1 clone1	652	*S. hjorti*	99.7	KY973332	Gjerde et al. (2017b)	*S. hjorti*	MT737809
	D1 clone2-D1 clone5	662–664	*S. venatoria/S. iberica*	98.9–99.9	KY973318, KY973321, KY973324, KY973325, KY973327	Gjerde et al. (2017b)	*Sarcocystis* sp.	MT737810- MT737813
Deer 2	D2 clone1-D2 clone5	662–666	*S. venatoria/S. iberica*	99.1–99.7	KY973318, KY973321, KY973324, KY973325, KY973327	Gjerde et al. (2017b)	*Sarcocystis* sp.	MT737814- MT737818
Deer 3	D3 clone5-D3 clone9	654	*S. hjorti*	99.7–100	GQ250990	Dahlgren and Gjerde (2010a)	*S. hjorti*	MW019997- MW020000
Deer 4	D4 PCR	654	*S. hjorti*	100	GQ250990	Dahlgren and Gjerde (2010a)	*S. hjorti*	MT737819
	D4 clone2-D4 clone3	666	*S. venatoria/S. iberica*	99.3–99.7	KY973321, KY973327	Gjerde et al. (2017b)	*Sarcocystis* sp.	MT737820,
								MT737821
	D4 clone4-D4 clone5	652–654	*S. hjorti*	99.5–99.7	KF831294, KY973332	(Gjerde, 2014a; Gjerde et al., 2017b)	*S. hjorti*	MT737822, MT737823
Deer 5	D5 PCR	630	*S. hjorti*	100	GQ250990	Dahlgren and Gjerde (2010a)	*S. hjorti*	MT737824
	D5 clone1-D5 clone5	652–654	*S. hjorti*	98.9–100	KY973332, KF831294	(Gjerde, 2014a; Gjerde et al., 2017b)	*S. hjorti*	MT737825- MT737829
Deer 6	D6 PCR	630	*S. ovalis*	100	GQ250988	Dahlgren and Gjerde (2010a)	*S. ovalis*	MT737830
	D6 clone1-	652–654	*S. ovalis*	99.2–100	LC184601	Irie et al. (2017)	*S. ovalis*	MT737831- MT737835
	D6 clone5							
Deer 7	D7 PCR	628	*S. hjorti*	100	GQ250990	Dahlgren and Gjerde (2010a)	*S. hjorti*	MT737836
	D7 clone1-D7 clone5	652–654	*S. hjorti*	99.2–100	KY973332, KF831294	(Gjerde, 2014a; Gjerde et al., 2017b)	*S. hjorti*	MT737837- MT737840
Deer 8	D8 clone6-D8 clone10	662–666	*S. venatoria/S. iberica*	99.4–99.9	KY973318, KY973321, KY973324, KY973327	Gjerde et al. (2017b)	*Sarcocystis* sp.	MW020001- MW020004
Deer 9	D9 clone2- D9 clone 6	662–666	*S. venatoria/S. iberica*	99.3–99.7	KY973318, KY973321, KY973323, KY973325, KY973327	Gjerde et al. (2017b)	*Sarcocystis* sp.	MW020005- MW020008
Deer 10	D10 clone1-D10 clone3, D10 clone5	662–664	*S. venatoria/S. iberica*	99.4–99.9	KY973318, KY973324, KY973325	Gjerde et al. (2017b)	*Sarcocystis* sp.	MT737841- MT737843, MT737845
	D10 clone4	652	*S. hjorti*	100	KY973332	Gjerde et al. (2017b)	*S. hjorti*	MT737844
Deer 11	D11 clone1	664	*S. venatoria/S. iberica*	99.1–99.4	KY973318, KY973325	Gjerde et al. (2017b)	*Sarcocystis* sp.	MT737846
	D11 clone2,	660	*S. linearis/S. taeniata*	99.4–100	KY973372, KU753890	(Prakas et al., 2016; Gjerde et al., 2017b)	*Sarcocystis* sp.	MT737847, MT737849, MT737850
	D11 clone4,							
	D11 clone5							
	D11 clone3	654	*S. hjorti*	99.4	KF831294	Gjerde (2014a)	*S. hjorti*	MT737848
Deer 12	D12 clone1-D12 clone5	664	*S. venatoria/S. iberica*	99.4–99.9	KY973318, KY973325	Gjerde et al. (2017b)	*Sarcocystis* sp.	MT737851- MT737854
Deer 13	D13 clone1-D13 clone3,	658–660	*S. linearis/S. taeniata*	97.9–99.9	KY973371, KY973372, KU753890, KT626602, MN334301, KF831293	(Gjerde, 2014a; Prakas et al., 2016; Reissig et al., 2016; Gjerde et al., 2017b; Rudaitytė-Lukošienė et al., 2020)	*Sarcocystis* sp.	MT737855- MT737857, MT737859
	D13 clone 5							
	D13 clone4	654	*S. hjorti*	99.2	KF831294	Gjerde (2014a)	*S. hjorti*	MT737858
Deer 14	D14 PCR	529	*S. pilosa*	100	LC466183	Irie et al. (2019)	*S. pilosa*	MT737860
	D14 clone1	653	*S. pilosa*	99.7	LC466183	Irie et al. (2019)	*S. pilosa*	MT737861
	D14 clone2,	654	*S. hjorti*	98.9–99.9	KF831294	Gjerde (2014a)	*S. hjorti*	MT737862, MT737865
	D14 clone5							
	D14 clone3,	664	*S. venatoria/S. iberica*	98.6–99.4	KY973318, KY973325	Gjerde et al. (2017b)	*Sarcocystis* sp*.*	MT737863, MT737864
	D14 clone4							
Deer 15	D15 clone1-D15 clone5	654	*S. hjorti*	99.7–100	KF831294	Gjerde (2014a)	*S. hjorti*	MT737866- MT737869
Deer 16	D16 PCR	654	*S. hjorti*	100	GQ250990	Dahlgren and Gjerde (2010a)	*S. hjorti*	MT737870
Deer 17	D17 clone1-D17 clone5	652–654	*S. hjorti*	99.7–100	KY973332, KF831294	(Gjerde, 2014a; Gjerde et al., 2017b)	*S. hjorti*	MT737871- MT737874
Deer 18	D18 clone1	660	*S. linearis/S. taeniata*	98.8–99.2	KY973372, KU753890	(Prakas et al., 2016; Gjerde et al., 2017b)	*Sarcocystis* sp*.*	MT737875
	D18 clone2-D18 clone5	662–666	*S. venatoria/S. iberica*	99.5–100	KY973318, KY973321, KY973324, KY973327	Gjerde et al. (2017b)	*Sarcocystis* sp*.*	MT737876- MT737879
Deer 19	D19 clone1,	664–666	*S. venatoria/S. iberica*	99.3–99.9	KY973318, KY973323, KY973325, KY973327	Gjerde et al. (2017b)	*Sarcocystis* sp*.*	MT737880, MT737882- MT737884
	D19 clone3- D19 clone5							
	D19 clone2	653	*S. pilosa*	99.9	LC466183	Irie et al. (2019)	*S. pilosa*	MT737881
Deer 20	D20 PCR	618	*S. hjorti*	100	GQ250990	Dahlgren and Gjerde (2010a)	*S. hjorti*	MT737885
	D20 clone1, D20 clone2, D20 clone4	662–664	*S. venatoria/S. iberica*	99.4–100	KY973318, KY973324, KY973325	Gjerde et al. (2017b)	*Sarcocystis* sp*.*	MT737886, MT737887, MT737889
	D20 clone3,	652–654	*S. hjorti*	99.7–100	KF831294, KY973332	(Gjerde, 2014a; Gjerde et al., 2017b)	*S. hjorti*	MT737888, MT737890
	D20 clone5							
Deer 21	D21 PCR	636	*S. hjorti*	100	GQ250990	Dahlgren and Gjerde (2010a)	*S. hjorti*	MT737891
	D21 clone1-D21 clone5	652–654	*S. hjorti*	99.5–100	KF831294, KY973332	(Gjerde, 2014a; Gjerde et al., 2017b)	*S. hjorti*	MT737892- MT737895
Deer 22	D22 clone1-D22 clone5	651–654	*S. hjorti*	99.5–100	KF831294, KY973332	(Gjerde, 2014a; Gjerde et al., 2017b)	*S. hjorti*	MT737896- MT737899
Deer 23	D23 clone2-D23 clone5	652–654	*S. hjorti*	99.7–99.9	KF831294, KY973332	(Gjerde, 2014a; Gjerde et al., 2017b)	*S. hjorti*	MT737900- MT737903
Deer 24	D24 clone1-D24 clone4	652–654	*S. hjorti*	99.9–100	KF831294, KY973332	(Gjerde, 2014a; Gjerde et al., 2017b)	*S. hjorti*	MT737904- MT737907
Deer 25	D25 clone1, D25 clone3	664	*S. venatoria/S. iberica*	99.3–99.7	KY973318, KY973325	Gjerde et al. (2017b)	*Sarcocystis* sp*.*	MT737908, MT737910
	D25 clone2,	654	*S. hjorti*	99.4.99.9	KF831294, KY973332	(Gjerde, 2014a; Gjerde et al., 2017b)	*S. hjorti*	MT737909, MT737911, MT737912
	D25 clone4,							
	D25 clone5							
Deer 26	D26 clone1, D26 clone3-D26 clone5	662–666	*S. venatoria/S. iberica*	99.4–99.7	KY973318, KY973321, KY973324, KY973327	Gjerde et al. (2017b)	*Sarcocystis* sp.	MW020010- MW020013
	D26 clone2	654	*S. hjorti*	99.9	GQ250990	Dahlgren and Gjerde (2010a)	*S. hjorti*	MW020009
Deer 29	D29 clone1,	636–637	*S. silva*	99.7	KY019056, KY019065	Gjerde et al. (2017a)	*S. silva*	MT737913, MT737916
	D29 clone4							
	D29 clone2,	648	*S. silva*	98.3–98.6	KY019059, KY019067	Gjerde et al. (2017a)	*Sarcocystis* sp*.*	MT737914, MT737915
	D29 clone3							
	D29 clone5	664	*S. venatoria/S. iberica*	98.8–99.1	KY973318, KY973325	Gjerde et al. (2017b)	*Sarcocystis* sp*.*	MT737917
Deer 30	D30 clone1-D30 clone5	664	*S. venatoria/S. iberica*	99.1–99.9	KY973318, KY973323, KY973324, KY973325, KY973327	Gjerde et al. (2017b)	*Sarcocystis* sp*.*	MT737918- MT737922
Deer 32	D32 clone1-D32 clone5	654	*S. hjorti*	99.7–99.9	KF831294	Gjerde (2014a)	*S. hjorti*	MT737923- MT737926
Deer 33	D33 clone1-D33 clone4	652–654	*S. hjorti*	99.7–99.9	KF831294, KY973332	(Gjerde, 2014a; Gjerde et al., 2017b)	*S. hjorti*	MT737927, MT737928
Deer 34	D34 clone1-D34 clone5	652–654	*S. hjorti*	99.1–99.9	KF831294, KY973332	(Gjerde, 2014a; Gjerde et al., 2017b)	*S. hjorti*	MT737929- MT737931

The obtained sequences were assigned to a determined *Sarcocystis* species if: (i) they showed more than 99% unambiguous BLASTn identity with GenBank® entries for which the species was known; (ii) the reference GenBank® sequences were supported by morphological data; (iii) the obtained sequences clustered with the reference GenBank® sequences in a phylogenetical tree (see below). Sequences showing >99% similarity with GenBank® sequences of more than one named *Sarcocystis* species (as it is generally the case between *S. linearis* and *S. taeniata* or between *S. venatoria* and *S. iberica* and sometimes also between *S. tenella and S. capracanis*) were recorded as *Sarcocystis* sp. The highest homology for each species was indicated in Table 2 and Supplementary Table 4. Sequences with less than 99% identity with named GenBank® entries were recorded as *Sarcocystis* sp. To assess the relationship of the sequences among them and with reported reference GenBank® sequences, a phylogenetic tree was built using the neighbour-joining method with the software Geneious R10 (https://www.geneious.com) (Supplementary Fig. 1). For this analysis, the obtained sequences were trimmed from the primer binding regions. Nucleotide and haplotype diversity within species of *Sarcocystis* was calculated using the DnaSP v6 software (Rozas et al., 2017).

## 3 Results

### 3.1 Histopathological examination of muscle samples from red deer

**Fig. 2:**
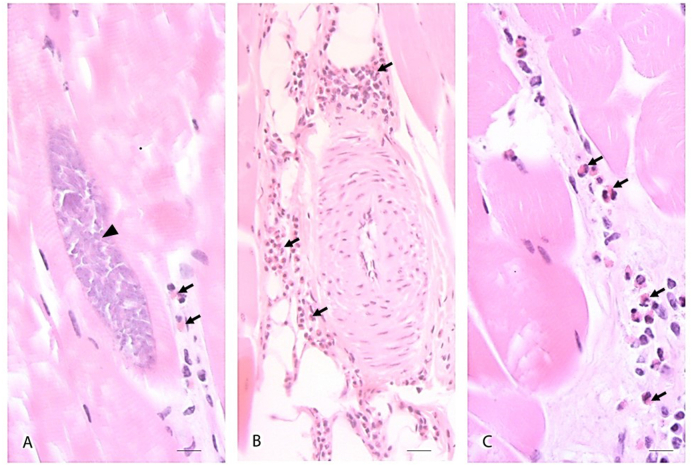
A–C: Extensive diffuse eosinophilic infiltration along connective tissue in fascia and skeletal fore limb muscle from a red deer showing grey-greenish discolouration of the carcass (Deer 7), characterized by eosinophilic leukocytes (black arrows) and lymphocytes; A: *Sarcocystis* cyst (“sarcocyst”)(arrowhead) and eosinophilic leucocytes (black arrows), bar = 10 μm. B: intramuscular blood vessel with hypertrophied media, bar = 20 μm. C: intramuscular eosinophilic infiltration (black arrows) along myofibers, bar = 10 μm. Haematoxylin and eosin (HE) staining. (For interpretation of the references to colour in this figure legend, the reader is referred to the Web version of this article.)

Histologically, inflammatory changes characterized by eosinophilic myositis and eosinophilic lymphoplasmacellular fasciitis were diagnosed in 18 (69%) and 17 (65%) out of 26 examined deer showing a greenish discolouration of the carcass, respectively, and sarcocysts were observed in 23 (89%) of these animals (i.e. in 23/26 and 9/13 limb and diaphragm meat samples, respectively) (Fig. 2). Besides, the media of many veins were significantly thickened by hypertrophy and hyperplasia. The endothelial cells were flat or cuboidal and the lumen of some vessels was almost completely occluded (Fig. 2). Meat samples from animals with normal carcass appearance (control group from location Rueun, *n* = 8) did not show inflammatory changes and *Sarcocystis* was detected in only 1/8 (13%) of the limb samples. When the histopathological results of all 47 analysed muscle samples (i.e. 34 limb samples and 13 diaphragm samples) from red deer with and without macroscopical carcass changes were considered together, a positive association between the presence of sarcocysts and eosinophilic myositis was found (Table 1). Sarcocysts were more frequently detected in muscle samples in which eosinophilic myositis was observed (84.6% out of 26), than in those samples without inflammatory changes (52.4% out of 21) (Fisher’ exact test *p* = 0.0250). Detailed results of the histopathological examination are indicated in Table 1.

Microscopical examination of faecal and intestine samples from red foxes and faecal samples from hunting dogs.

By flotation in sucrose solution, *Sarcocystis* oocysts and/or sporocysts were microscopically detected in 10% (11/106) of the faecal samples and 30% (6/20) of the samples from the intestinal mucosa from red foxes (Supplementary Table 2), and in the faeces from two (Dog 5 and Dog 6) (16.7%) out of 12 hunting dogs (Supplementary Table 3). Dog 5 was a female, 9-month-old Hanover Hound and Dog 6 was a male, 13-year-old, Magyar Vizsla. Both dogs were used for hunting purposes in the Canton of Grisons and were fed with fresh raw meat and heart from red deer and other hunted animals, besides receiving commercial feed.

Molecular diagnosis of *Sarcocystis* infection in red deer, red foxes, and hunting dogs.

Positive PCR results for *Sarcocystis* spp. were obtained in muscle samples from all 26 (100%) analysed red deer showing a grey-greenish discolouration of the carcass (Table 1). Direct sequencing and cloning of the obtained PCR products in a plasmid vector followed by sequencing allowed molecular species discrimination in eight and 25 animals, respectively (Table 1, Table 2). At least five different *Sarcocystis* species could be identified by molecular methods: *S. hjorti*, *S. venatoria/S. iberica*, *S. linearis/S. taeniata*, *S. pilosa* and *S. ovalis* (Table 2). *Sarcocystis hjorti* was the most frequently detected species in 19 (73%) of the deer, followed by *S. venatoria/S. iberica* in 14 (54%) animals, *S. linearis/S. taeniata* in three (12%), *S. pilosa* in two (8%) and *S. ovalis* in one (4%) animal. By cloning of the PCR products, one sole *Sarcocystis* species was detected in 14 of the animals (i.e. *S. hjorti n* = 9; *S. venatoria/S. iberica n* = 4 and *S. ovalis n* = 1). In three red deer (Deer 4, 14 and 20), in which direct sequencing indicated infection by only one species, the cloning technique revealed infection with two to three different *Sarcocystis* species (including the *Sarcocystis* species determined by direct sequencing) (Table 2), showing the higher diagnostic sensitivity of cloning over direct sequencing. Co-infections with two and three different *Sarcocystis* species were detected in nine and two animals, respectively (Table 1, Table 2 and Supplementary Table 4). In one of the samples (Deer 16), no further *Sarcocystis* species identification by cloning could be performed. While *Sarcocystis* DNA could be detected in muscle samples from 100% (26/26) of the animals with greenish macroscopic changes in the muscles, only 62.5% (5/8) of the samples from animals with normal carcass appearance yielded positive PCR results (Fisher's exact test, *p* = 0.009). Direct sequencing did not allow species differentiation in any of the five animals. By cloning, sequences corresponding to *S. hjorti* and *S. venatoria/S. iberica* were detected in three and one animal, respectively. A further animal showed molecular evidence of co-infection with *S. venatoria/S. iberica* and *S. silva* (Deer 29) (Table 1, Table 2).

In red foxes, the presence of *Sarcocystis* DNA could be confirmed by PCR and sequencing in parasites isolated from 7/11 faecal samples and in 6/6 samples from intestine mucosa, in which *Sarcocystis* sporocysts/oocysts had been microscopically detected. By direct sequencing, amplicons obtained from 11 of the foxes showed 100% identity with GenBank® sequences of *S. tenella* (*n* = 7), *S. gracilis* (*n* = 2), *S. hjorti* (*n* = 1) or *S. miescheriana* (*n* = 1)(Table 3, Supplementary Table 5). Further cloning and sequencing of these PCR products allowed the confirmation of the results in all nine tested foxes (samples from Foxes 46 and 115 could not be cloned) and also revealed the presence of co-infection with further non-defined *Sarcocystis* species in six of these animals. These sequences were mainly related to *S. capracanis/S. tenella* (93.4–98.9% sequence identity) in Foxes 1, 10, 108, 112, 120 and 126, and to *S. hircicanis/S. arieticanis* (94.5–95.2% sequence identity) in Fox 108. These sequences were deposited in GenBank® as *Sarcocystis* sp. (Table 3, Supplementary Table 5, Supplementary Fig. 1).

**Table 3:** Description of *Sarcocystis* spp. 18S rRNA gene sequences amplified from *Sarcocystis* oocysts/sporocysts from faeces and intestinal mucosa of red foxes or faeces of hunting dogs from Grisons, Switzerland, obtained by direct sequencing of the PCR products or after cloning into vector plasmids. Samples from Foxes 2, 12, 33 and 98 yielded negative PCR results for *Sarcocystis*.

Animal ID	Sequence ID	Sequence length (bp)	BLASTn identity	(%)	GenBank® accession no. (reference sequence)	Reference	*Sarcocystis* sp. (this study)	GenBank® accession no. (this study)
Fox 1	Fox1 PCR	623	*S. tenella*	100	KP263759	Kolenda et al. (2015)	*S. tenella*	MT737932
	Fox1 clone1,	646–647	*S. tenella*	99.7–99.9	MK420019, KP263759	(Kolenda et al., 2015; Gjerde et al., 2020)	*S. tenella*	MW020893, MW020894, MW020896, MW020897
	Fox1 clone2,							
	Fox1 clone4,							
	Fox1 clone5							
	Fox1 clone3	642	*S. capracanis*	93.8	KU820983	Hu et al. (2016)	*Sarcocystis* sp.	MW020895
			*S. tenella*	93.4	MK420019	Gjerde et al. (2020)		
Fox 6	Fox6 clone1, Fox6 clone5	647	*S. capracanis/S. tenella*	98.5–99.9	KU820983, MF039329,	(Jeffries et al., 1997; Hu et al., 2016;	*Sarcocystis* sp.	MT737943, MT737947
					L76472, MK420019	Hu et al., 2017; Gjerde et al., 2020)		
	Fox6 clone2-Fox6 clone4	652–654	*S. hjorti*	99.5–100	KY973332, KF831294	(Gjerde, 2014a; Gjerde et al., 2017b)	*S. hjorti*	MT737944- MT737946
Fox 10	Fox10 PCR	625	*S. tenella*	100	KP263759	Kolenda et al. (2015)	*S. tenella*	MT737933
	Fox10 clone1	646	*S. capracanis/S. tenella*	98.3–98.5	KU820983; MF039329	(Hu et al. 2016, 2017)	*Sarcocystis* sp.	MW020898
	Fox10 clone3,	647–648	*S. tenella*	99.2–100	MF039329, KP263759	(Kolenda et al., 2015; Hu et al., 2017)	*S. tenella*	MW020899, MW020900
	Fox10 clone4							
Fox 15	Fox15 clone1- Fox15 clone4	645–649	*S. capracanis/S. tenella*	97.1–99.7	KU820982, KU820983, KP263759, MK420018	(Kolenda et al., 2015; Hu et al. 2016, 2017; Gjerde et al., 2020)	*Sarcocystis* sp.	MT737948- MT737951
	Fox15 clone5	642	*S. hircicanis/S. aretiecanis*	95.8–96.3	KU820984; MK420017	(Hu et al., 2016; Gjerde et al., 2020)	*Sarcocystis* sp.	MT737952
Fox 16	Fox16 PCR	639	*S. miescheriana*	100	MH404232	Gazzonis et al. (2019)	*S. miescheriana*	MT737934
	Fox16 clone1-Fox16 clone8	664	*S. miescheriana*	99.9–100	MH404232	Gazzonis et al. (2019)	*S. miescheriana*	MW020901- MW020903
Fox 46	Fox46 PCR	617	*S. hjorti*	100	GQ250990	Dahlgren and Gjerde (2010a)	*S. hjorti*	MT737935
Fox 47	Fox47 PCR	638	*S. gracilis*	100	MN334289	Rudaitytė-Lukošienė et al. (2020)	*S. gracilis*	MT737936
	Fox47 clone1-Fox47 clone4	675	*S. gracilis*	99.7–100	MN334289	Rudaitytė-Lukošienė et al. (2020)	*S. gracilis*	MW020904- MW020907
	Fox47 clone5	647	*S. tenella*	99.9	KP263756	Kolenda et al. (2015)	*S. tenella*	MW020908
Fox 108	Fox108 PCR	624	*S. tenella*	100	KP263759	Kolenda et al. (2015)	*S. tenella*	MT737937
	Fox108 clone1	647	*S. capracanis/S. tenella*	98.8–98.9	KU820983, MF039329	(Hu et al. 2016, 2017)	*Sarcocystis* sp.	MW020909
	Fox108 clone2, Fox108 clone3	651	*S. hircicanis/S. aretiecanis*	94.5–95.2	KU820984, MK420017	(Hu et al., 2016; Gjerde et al., 2020)	*Sarcocystis* sp.	MW020910, MW020911
	Fox108 clone4,	647	*S. tenella*	99.7–99.9	MK420019	Gjerde et al. (2020)	*S. tenella*	MW020912
	Fox108 clone5							
Fox 112	Fox112 PCR	621	*S. tenella*	100	MK420019	Gjerde et al. (2020)	*S. tenella*	MT737938
	Fox112 clone 4 Fox112 clone 5, Fox112 clone 7,	647–648	*S. tenella*	99.4–99.5	MK420019, KP263759,	(Kolenda et al., 2015; Hu et al., 2017; Gjerde et al., 2020)	*S. tenella*	MW020914, MW020915, MW020917, MW020918
	Fox112 clone 8				MF039329			
	Fox112 clone 6	647	*S. capracanis/S. tenella*	98.6–98.9	KU820983, KP263759	(Kolenda et al., 2015; Hu et al., 2016)	*Sarcocystis* sp.	MW020916
Fox 115	Fox115 PCR	637	*S. gracilis*	100	MN334289	Rudaitytė-Lukošienė et al. (2020)	*S. gracilis*	MT737939
Fox 120	Fox120 PCR	621	*S. tenella*	100	KP263759	Kolenda et al. (2015)	*S. tenella*	MT737940
	Fox120 clone1,	647	*S. tenella*	99.2–99.7	MF039329, KP263756,	(Kolenda et al., 2015; Hu et al., 2017)	*S. tenella*	MW020919, MW020920, MW020922
	Fox120 clone7,				KP263759			
	Fox120 clone9							
	Fox120 clone8, Fox120 clone10	645–647	*S. tenella/S. capracanis*	98.6–98.9	KP263756, KP263759	(Kolenda et al., 2015; Hu et al., 2016)	*Sarcocystis* sp.	MW020921, MW020923
				98.8	KU820982, KU820983			
Fox 125	Fox125 PCR	624	*S. tenella*	100	KP263759	Kolenda et al. (2015)	*S. tenella*	MT737941
	Fox125 clone1-Fox125 clone7	647	*S. tenella*	99.1–100	MF039329, MK420019	(Hu et al., 2017; Gjerde et al., 2020)	*S. tenella*	MW021141- MW021143
Fox 126	Fox126 PCR	622	*S. tenella*	100	KP236759	Kolenda et al. (2015)	*S. tenella*	MT737942
	Fox126 clone5,	647	*S. tenella*	99.2–99.4	MF039329, KP263758	(Kolenda et al., 2015; Hu et al., 2017)	*S. tenella*	MW020924, MW020927
	Fox126 clone9							
	Fox126 clone6, Fox126 clone8	642–647	*S. capracanis/S. tenella*	93.2–98.5	KU820983, MF039329, MK420019	(Hu et al. 2016, 2017; Gjerde et al., 2020)	*Sarcocystis* sp.	MW020925, MW020926
Dog 5	Dog5 clone1,	663	*S. capreolicanis*	99.4–99.5	KY019029, MN334253	(Gjerde et al., 2017a; Rudaitytė-Lukošienė et al., 2020)	*S. capreolicanis*	MT737953, MT737956
	Dog5 clone5							
	Dog5 clone2, Dog5 clone3	675	*S. gracilis*	99.1–99.4	MN334289	Rudaitytė-Lukošienė et al. (2020)	*S. gracilis*	MT737954, MT737955
Dog 6	Dog6 clone1, Dog6 clone3	656	*S. linearis/S. taeniata*	98.7–99	MN334294, KU753890	(Prakas et al., 2016; Rudaitytė-Lukošienė et al., 2020)	*Sarcocystis* sp.	MT737957, MT737958
	Dog6 clone9	675	*S. gracilis*	99.8	MN334289	Rudaitytė-Lukošienė et al. (2020)	*S. gracilis*	MT737959

In two further foxes (Fox 6 and Fox 15), direct sequencing suggested co-infection with more than one *Sarcocystis* species (Table 1) and cloning was performed. In one of these foxes (Fox 6), sequencing of the obtained clones revealed co-infection by *S. hjorti,* and *S. tenella/S. capracanis*. In the other fox (Fox 15), sequences of *S. capracanis/S. tenella* and a non-defined *Sarcocystis* sp. (Fox15 clone5) with 96.5% and 96.1% sequence identity with GenBank® sequences of *S. hircicanis* and *S. arieticanis*, respectively, were observed (Table 3, Supplementary Table 5).

Four out of 11 faecal samples in which *Sarcocystis* sporocysts had been observed after flotation were negative by PCR. These samples contained only very few sporocysts.

Both samples from hunting dogs, in which sporocysts of *Sarcocystis* spp. had been observed after flotation, were positive by PCR. By direct sequencing, a *Sarcocystis* mixed infection was assumed for both samples; therefore, these PCR products were cloned. In Dog 5, sequencing of the obtained clones allowed the detection of sequences with 99.1–99.4% and 99.4–99.6% identity with GenBank® sequences of *S. gracilis* and *S. capreolicanis,* respectively. In Dog 6, sequences with 99.9% identity with GenBank® sequences of *S. gracilis* and 99.1 and 98.9% % identity with *S. linearis* and *S. taeniata*, respectively, were found (Table 3).

Sequence analysis revealed high intraspecific variability in the 18S rRNA gene sequence obtained from *Sarcocystis* from all three different hosts. The number of isolates, number of identified variants for each *Sarcocystis* species, and further parameters of intraspecific genetic variability are shown in Table 4. A phylogenetic tree inferred using the neighbour-joining method shows the relationship of the obtained 18S rRNA partial sequences with reference *Sarcocystis* sequences annotated in GenBank® (Supplementary Fig. 1).

**Table 4:** Parameters of intraspecific genetic variability in the 18S rRNA gene in *Sarcocystis* species or groups of closely related species detected in red deer, red foxes and hunting dogs from Grisons, Switzerland.

*Sarcocystis* species	h/n	Hd	Identity (%)	S	π
*S. hjorti*	49/74	0.897	98.01–100	84	0.00416
*S. tenella*	21/31	0.882	99.9–100	66	0.00835
*S. venatoria/S.iberica*	44/55	0.962	97.6–100	78	0.00480
*S. linearis/S.taeniata*	10/10	1	95.7–99.7	35	0.01497
*S. ovalis*	4/6	0.8	98.93–99.85	7	0.00372
*S. pilosa*	3/3	1	99.54	3	0.000378
*S. silva*	4/4	1	96.8–98.9	11	0.00868
*S. gracilis*	7/9	0.917	98.81–99.26	17	0.00619
*S. capreolicanis*	2/2	1	99.8%	1	0.00151
*S. capracanis/S. tenella*	5/10	0.667	98.9–100	6	0.00294
*S*. *miescheriana*	3/5	0.7	99.9–100	3	0.00126
Total	152/209				

## 4 Discussion

Grey-greenish discolouration in meat has been attributed to several causes, such as eosinophilic inflammatory myopathies, and post-mortem microbial and non-microbial enzymatic processes leading to the production of hydrogen sulphide and alterations in the myoglobin pigments of striated muscles (Stephan et al. 1997, 2012). Besides, infections with *Onchocerca* spp. (Filarioidea; Onchocercidae) nematodes were associated with greenish discolouration and oedema of subcutaneous tissues in cervids (Laaksonen et al., 2017) and domestic species (Solismaa et al., 2008). Adult *Onchocerca* worms are localised free or in nodules in subcutaneous tissues and produce microfilariae in these sites (Bosch et al., 2016; Boijsen et al., 2017). Dead or dying adult worms or microfilariae may trigger inflammatory reactions characterized by infiltration of eosinophilic granulocytes and multifocal nodular lymphoplasmacytic aggregations around them, followed by calcification and fibrosis (Solismaa et al., 2008). Four *Onchocerca* species were described in red deer in Central Europe (i.e. *O. skrjabini* and *O. garmsi* free in subcutaneous tissues, and *O. flexuosa* and *O. jakutensis* in subcutaneous nodules) (Bosch et al., 2016; Boijsen et al., 2017), and *O. jakutensis* infection was reported in 26% of red deer of the Grisons region (Bosch et al., 2016). Although the occurrence of *Onchocerca* spp. infections, as well as their putative involvement in the development in greenish discolouration in some of the carcasses in the present study cannot be completely ruled out, we have neither observed macroscopic nodules, nor the presence of adult worms or microfilariae in any of the histological slides analysed.

In our study, histopathological examination revealed eosinophilic myositis and/or eosinophilic lymphoplasmacellular fasciitis as the underlying cause for the observed macroscopic changes in 73% of the analysed animals, and *Sarcocystis* infection was detected in 89 and 100% of these samples by histopathology or molecular analysis, respectively (Table 1). Eosinophilic myositis is an inflammatory condition of striated skeletal and cardiac muscle, mainly characterized by infiltration with eosinophils, followed by myocytes degeneration and building of granulomas at later stages (Dubey et al., 2016). The observed thickening of the vessel walls could be related to the parasite multiplication in the endotelial cells, which may trigger a hypertrophic reaction; however, no parasites could be observed associated with these changes and further investigation is needed to support this hypothesis. Affected animals are in most cases asymptomatic and the pathology is first detected at the abattoir level, leading to carcass condemnation (Dubey et al., 2016). Several studies provided evidence for a causal association between *Sarcocystis* spp. infection and eosinophilic myositis in different animal species such as cattle (Gajadhar et al., 1987; Wouda et al., 2006; Aráoz et al., 2019; Jensen et al., 1986; Vangeel et al., 2013), sheep (Jensen et al., 1986), horses (Herd et al., 2015) and alpacas (La Perle et al., 1999), including the experimental reproduction of the lesions in cattle (Vangeel et al., 2012). However, the details of the pathological mechanisms of sarcosporidiosis causing eosinophilic myositis/fasciitis and grey-greenish tissue discolouration are mostly unknown. Possible triggers for the immune response may be the release of antigens after rupture of the sarcocyst wall (Jensen et al., 1986; Gajadhar and Marquardt, 1992; Vangeel et al., 2012), as well as hypersensitivity mechanisms (Granstrom et al., 1989). *Sarcocystis* antigens (lysed sarcocysts) inoculated intramuscularly in cattle were shown to induce local lesions at the injection site, characterized by massive infiltration of eosinophilic granulocytes, reactive macrophages, T-cells and B-cells, resembling natural eosinophilic myositis (Vangeel et al., 2012). Besides, a genetical predisposition of some individual animals was also suggested to play a role in the pathogenesis of eosinophilic myositis (Herd et al., 2015; Granstrom et al., 1989). This hypothesis would be in agreement with the low prevalence of eosinophilic myositis despite the high prevalence of *Sarcocystis* infection in certain animal species like cattle (Aráoz et al., 2019; Vangeel et al., 2013). Observation of damaged intralesional sarcocysts in histologic sections has been an argument in favour of the role of these parasites in the pathogenesis of eosinophilic myopathy (Vangeel et al., 2013; Gajadhar and Marquardt, 1992; Wouda et al., 2006). In this study, sarcocysts were only seldom detected in the middle of the lesions but were frequently observed in the surrounding areas. However, it must be considered that the sensitivity of histological examination for the detection of *Sarcocystis* is limited (Jensen et al., 1986), and that the specific immune response against this parasite and its subsequent destruction could also account for a decreased histological detection of sarcocysts in the lesions (Vangeel et al., 2013; Gajadhar and Marquardt, 1992). Sarcocysts were detected in only one out of eight meat samples with normal appearance by microscopy; however, when DNA extraction was performed on 0.5 g meat samples (a larger sample than that analysed by histology), *Sarcocystis* DNA could be detected in five of those samples. Therefore, the presence of a *Sarcocystis* infection could not be ruled out in the control animals, but we can presume that the density of tissue cysts was lower than in animals with greenish carcass discoloration. Considering only samples from limb muscle (as these were tested histopathologically in all 34 animals in the study), at least one sarcocyst was observed in H&E stained sections from 23 out of 26 (89%) animals with macroscopic carcass changes and in only in 1 out of 8 (13%) of the samples from animals with normal carcass appearance (Fisher's exact test, *p* = 0.000056), suggesting a higher parasite burden in animals with pathological changes.

This study revealed that red deer in the canton Grisons may serve as an IH for at least six *Sarcocystis* species. *Sarcocystis hjorti* was the most frequently detected *s*pecies in red deer showing a grey-greenish discolouration of the carcass, and it had been reported in animals showing this pathology in this region before (Stephan et al., 2012). However, also other *Sarcocystis* species (i.e. *S. venatoria/S. iberica, S. linearis/S. taeniata, S. pilosa* and *S. ovalis*) have been now detected, suggesting that different *Sarcocystis* species might be involved in the pathogenesis of eosinophilic fasciitis/myositis in red deer. Accordingly, a study in Belgium showed that four different *Sarcocystis* species were associated with eosinophilic myositis in slaughtered cattle (Vangeel et al., 2013).

To date, 11 different *Sarcocystis* species forming sarcocysts of five major morphological types have been described infecting European red deer (Gjerde et al., 2017b). Mixed natural infections with several *Sarcocystis* species seem to be very common in free-ranging cervids (Dahlgren and Gjerde, 2010a). Accordingly, we found evidence of co-infection with two or more *Sarcocystis* species in 11 of the analysed red deer with macroscopical changes in the carcass, and in one of the animals of the control group (Table 2). Due to the limited sample size of 0.5 g muscles/animal, the possible presence of co-infections in the remaining animals cannot be ruled out.

We have here relied on molecular methods for the identification of *Sarcocystis* species affecting red deer and putative DH foxes and dogs. Molecular studies are necessary because different *Sarcocystis* species affecting cervids have a highly similar cyst morphology by light and electron microscopy (Dahlgren and Gjerde, 2010a; Gjerde et al., 2017b; Abe et al., 2019a), and oocysts and sporocysts in the DH cannot be discriminated by microscopic techniques (Dubey et al., 2016). Several studies based the identification of *Sarcocystis* at the species level on the 18S ribosomal RNA gene sequence as it was performed in this study, but also the 28S rRNA gene, the ITS1 region, the mitochondrial cytochrome c oxidase subunit I gene (*cox*1) or different combinations of these targets have been used for this purpose, enhancing the diagnostic possibilities (Gjerde 2013, 2014b; Gjerde et al., 2017b; Moré et al., 2016; Dahlgren and Gjerde, 2010a; Cerqueira-Cézar et al., 2018). The methodology based on the 18S rRNA gene amplification with subsequent cloning and sequencing has been successfully used to identify co-infections with several *Sarcocystis* spp. and to gather information on potential DH of these parasites (Moré et al., 2016). Cloning is necessary because in the case of mixed infections, direct sequencing alone would fail to identify the species involved as we could also observe in the present study (Table 2, Table 3, Supplementary Tables 4 and 5). For discrimination between some closely related species affecting red deer such as *S. tarandi*/*S. elongata, S. venatoria*/*S. iberica* and *S. linearis/S. taeniata,* differences at the 18S rRNA region may not be enough, and the use of a further genetic marker such as *cox*1 may be needed (Gjerde, 2014b; Gjerde et al., 2017b). The same applies to certain *Sarcocystis* species affecting small ruminants such as *S. tenella* and *S. capracanis* (Moré et al., 2016). The procedure used in this study, based on extraction of DNA from large (0.5 g) muscle samples, had the advantage (vs. isolation of DNA from individual sarcocysts), that it allowed molecular diagnosis of coinfections with several *Sarcocystis* species or genotypes, and that both large and small-sized sarcocysts (which could have been probably missed during microscopic isolation of individual sarcocysts from fresh muscle) had similar chances to be present in the samples.

*Sarcocystis hjorti* was the most frequently detected species by molecular methods in Swiss red deer with grey-greenish discolouration of the carcass. It was observed in 19 out of 26 (73%) animals from all four sampled regions in Grisons. This was also the most frequent *Sarcocystis* species in Norwegian red deer, where a prevalence of 95% (35/37) was recorded (Dahlgren and Gjerde, 2010a). *Sarcocystis hjorti* was also reported from red deer in Lithuania (Prakas and Butkauskas, 2012) and Spain (Gjerde et al., 2017b), suggesting a widespread distribution in Europe. This species uses red deer and moose (*Alces*) as IH (Gjerde, 2014b; Prakas et al., 2019), and it was shown experimentally, that red foxes and arctic foxes (*Vulpes lagopus*) could act as DH of *S. hjorti* isolated from moose (Dahlgren and Gjerde, 2010b). In the present study, this parasite was also detected in two red foxes (Fox 6 and 46) from the same region, confirming that the red fox is a natural DH of *S. hjorti*.

The second most frequently observed species was the cluster *Sarcocystis venatoria/S. iberica* in 14 out of 26 (54%) animals from all four sampled regions (i.e. Davos, Rueun, Cunters and Filisur). These species were first described in recent years from red deer in Spain (Gjerde et al., 2017b), and this is the first record outside de Iberian Peninsula. The DH are unknown but canids were suggested (Gjerde et al., 2017b). No infection with *Sarcocystis venatoria/S. iberica* was recorded in foxes or dogs in this study.

In one of the analysed red deer (Deer 6), infection by *S. ovalis* was detected by both direct sequencing and cloning. This *Sarcocystis* species uses red deer, moose (Gjerde, 2014b; Prakas et al., 2019) and sika deer (Irie et al., 2017 Rudaitytė-Lukošienė et al., 2018) as IH and corvid birds as DH. So far, *S. ovalis* has been only detected in red deer from Norway (Dahlgren and Gjerde, 2010a; Gjerde, 2013), moose from Norway, Canada (Dahlgren and Gjerde, 2008) and Lithuania (Prakas et al., 2019) and sika deer from Japan (Irie et al., 2017) and Lithuania (Rudaitytė-Lukošienė et al., 2018), and this represents the first record of *S. ovalis* in Central Europe. The European magpie (*Pica*) and the Japanese jungle crow (*Corvus macrorhynchos*) have been confirmed as definitive hosts for *S. ovalis* (Gjerde and Dahlgren, 2010; Irie et al., 2017). However, in Europe, other corvid birds, such as the carrion crow (*Corvus cornix*) and the common raven (*Corvus corax*) are supposed to act as additional and possibly more important definitive hosts for *S. ovalis*, because they are the main corvid species feeding on carcasses of large animals (Gjerde and Dahlgren, 2010; Gjerde, 2014b; Gjerde and Dahlgren, 2010). Corvid birds are common in forest areas all over the canton Grisons and have a diverse diet, including carrion. We have found *S. ovalis* in only one out of 45 analysed muscle samples from red deer, suggesting that sarcocysts of *S. ovalis* were not present in high numbers in the samples, or that this species does not frequently occur in the region. A low frequency of infection was also observed in studies from Lithuania; in which *S. ovalis* was only detected in 2 out of 33 (6.4%) examined sika deer (Rudaitytė-Lukošienė et al., 2018). Accordingly, it was reported that *S. ovalis* and other *Sarcocystis* species using corvids as DH seem to produce only low-to moderate-intensity infections in the IH, in contrast to species being transmitted by canids (Gjerde and Dahlgren, 2010). This could be due to a restricted ability of the parasite to multiply in the IH, or to reduced environmental contamination through the DH, leading to infections with few sporocysts (Gjerde and Dahlgren, 2010).

In two red deer (Deer 14 and 19) from the same sampling region (i.e. Cunters), sequences with 100 and 99.9% identity with GenBank® sequences of *S. pilosa* from sika deer (*Cervus nippon*) LC466183 (Irie et al., 2019) were detected. *Sarcocystis pilosa* has been so far described infecting sika deer in Lithuania (Prakas et al., 2016) and Japan (Abe et al. 2019a, 2019b; Irie et al., 2019), but there are no previous records of infection in red deer. Our findings are highly suggestive that red deer could also act as IH of this parasite. Recently, red foxes have been found to serve as DH for *S. pilosa* in Japan (Irie et al., 2020).

In three further red deer (Deer 11, 13 and 18) from the same sampling region in Grisons mentioned above (i.e. Cunters), sequences with 99.2–100% identity with GenBank® entries of *S. linearis* derived from red deer (KY973371, KY973372) (Gjerde et al., 2017b) and roe deer (MN334301) (Rudaitytė-Lukošienė et al., 2020) were revealed by cloning. This recently described *Sarcocystis* species uses red deer (Gjerde et al., 2017b), roe deer (*Capreolus*) (Gjerde et al., 2017a; Rudaitytė-Lukošienė et al., 2020) and moose (Prakas et al., 2019) as IH. Its DH was still not described, but based on its phylogenetic position, canids were suspected to play this role (Gjerde et al., 2017a). It is to note, that *S. linearis* shares a high degree of identity (97.9–99.7%) at the 18S rRNA sequence with *S. taeniata* (Gjerde et al., 2017b), a *Sarcocystis* species infecting moose (Dubey et al., 2016), and it is not possible to unequivocally separate both species on the sole basis of this gene (Gjerde et al., 2017b). Moreover, it was postulated that reported 18S rRNA sequences of *Sarcocystis* from red deer in Lithuania (JN256126–JN256127) (Prakas et al., 2016) and Argentina (KT626602)(Reissig et al., 2016), originally attributed to *S. taeniata* may actually correspond to the new described species *S. linearis* (Gjerde et al., 2017b). Accordingly, also the sequences obtained in our study showed a high similarity (98.8–99.9% identity) with GenBank sequences annotated as *S. taeniata* (Table 2, Supplementary Table 4). Therefore, and as morphological information of individual sarcocysts was lacking, we have named these sequences as *S. linearis*/*S. taeniata* and annotated them as *Sarcocystis* sp. in GenBank®. Besides, we have provided a phylogenetical tree to show their relationship with other reported sequences (Supplementary Fig. 1). This shows that although the 18S rRNA gene marker has been widely used to differentiate *Sarcocystis* species it has some limitations. It has been also reported that *S. venatoria* and *S. iberica* may share an identity of 99.2–100% at the 18S rRNA sequence (Gjerde et al., 2017b). In our study we detected sequences 99–100% identical to GenBank® entries for *S. venatoria* but we have named these sequences as *S. venatoria*/*S. iberica* and annotated them as *Sarcocystis* sp., because the possibility that *S. iberica* could have been present in our sampling cannot be ruled out. A further genetic marker such as *cox*1 would be needed to unequivocally discriminate between these closely related species affecting red deer (Gjerde et al., 2017b).

*Sarcocystis* was detected by PCR in five out of eight red deer with normal carcass appearance. By cloning of the PCR products, three species could be identified: *S. hjorti* in three animals and *S. venatoria/S. iberica* in two other animals. Interestingly, sequences with 99.7% identity with GenBank sequences of *S. silva* (KY019065) were identified in one of the animals co-infected with *S. venatoria*/*S. iberica* (Deer 29). This is noteworthy, because *S. silva* had been so far only reported from moose and roe deer (Dubey et al., 2016), and this would represent the first record of this species in red deer. The DH of *S. silva* is still unknown, but based on the phylogenetic position of this species, they do not appear to be canids (Gjerde, 2012). Our molecular findings of *S. silva* and also *S. pilosa* in red deer would need further morphological investigation, to confirm if these species infect red deer, or if these findings represent other still not described *Sarcocystis* species with similar sequence homology.

As it was already reported for various *Sarcocystis* species affecting cervids (Gjerde, 2012; Gjerde et al. 2017a, 2017b; Rudaitytė-Lukošienė et al., 2020), a great intraspecific genetic variability at the 18S rRNA gene sequence level was also observed for *S. hjorti, S. venatoria/S. iberica, S. ovalis, S. pilosa, S. silva, S. capreolicanis* and S*. gracilis* in this study (Table 4).

Interestingly, no *Sarcocystis* species with felids as suspected DH based both on phylogenetical and epidemiological observations, such as *S. elongata, S. truncata* and *S. tarandi* (Gjerde, 2014b) were found in red deer in this study, suggesting that felids do not play a major role in the epidemiology of sarcocystosis in red deer in this region. This observation is supported by the fact that and only low numbers of wild felids such as lynxes are known to be present in the canton of Grisons (Bundesamt für Umwelt BAFU, 2016) and wild cats (*Felis silvestris*) are not supposed to occur in the region (https://www.wildtier.ch/projekte/wildkatzenmonitoring).

A further aim of the study was to investigate the involvement of red foxes and hunting dogs as definitive hosts of *Sarcocystis* species affecting red deer. Over the last few years, numerous studies have been performed to enlighten the life cycle of *Sarcocystis* affecting cervids (Dahlgren and Gjerde, 2010b; Irie et al. 2017, 2020; Gjerde and Dahlgren, 2010); however, for several species, the definitive hosts and many aspects about their epidemiology are still unknown. Due to the broad dietary habits, foxes may serve as DH for several *Sarcocystis* species using different IH (Moré et al., 2016). In this study, molecular typing of *Sarcocystis* oocysts/sporocysts isolated from faeces or intestinal mucosa of red foxes based on the 18S rRNA genetic marker allowed the identification of at least four *Sarcocystis* species: *S. tenella* (*n* = 8), *S. gracilis* (*n* = 2), *S. hjorti* (*n* = 2) and *S. miescheriana* (*n* = 1). These *Sarcocystis* species use either sheep and mouflon (*Ovis orientalis*)(*S. tenella*); roe deer (*S. gracilis),* red deer and moose (*S. hjorti*) or wild boars and pigs (*S. miescheriana*) as IH (Dubey et al., 2016), and their finding represents an indicator of the dietary habits of the foxes in the region. Besides, by cloning of PCR products, further sequences related to *S. capracanis/S. tenella* and *S. hircicanis/S. arieticanis* were found in eight of the foxes (Fox 1, 6, 10, 15, 108, 112, 120 and 126), which could not be assigned to a determined *Sarcocystis* species, and were annotated as *Sarcocystis* sp. (Table 3, Supplementary Table 5 and Supplementary Fig. 1). Eight of the obtained 18S rRNA sequences from foxes matched 100% with GenBank® sequences of *S. tenella* and thus they were annotated as such. However, *S. tenella* shares a high sequence homology with *S. capracanis* at this region, and the discrimination between these two species is sometimes difficult. Therefore, some sequences which showed >99% homology with both *S. tenella* and *S. capracanis* were annotated as *Sarcocystis* sp. (Table 3, Supplementary Table 5).

Observation of *S. hjorti* sporocysts in red foxes confirms previous experimental findings (Dahlgren and Gjerde, 2010b) and the role of this species as a definitive host for *S. hjorti* in nature. Sequence analysis of amplicons obtained from oocysts/sporocysts of several foxes revealed patent infection with different *Sarcocystis* species at the same time, showing the great epidemiological importance of the red fox in the environmental dissemination of these parasites.

In this study, intestine mucosa seemed to be a better matrix than faeces to detect *Sarcocystis* infection in DH. By microscopy, *Sarcocystis* oocysts/sporocysts were identified in 30% (6 out of 20) of intestine samples and only in 10.4% (11 out of 106) of faecal samples from red foxes. Accordingly, a higher prevalence of *Sarcocystis* infections in DH in studies using mucosal scrapings (vs faecal samples) had been previously observed (Moré et al., 2016). This could be related to a higher amount of *Sarcocystis* oocysts and sporocysts in mucosal scrapings than in faecal samples, and a lower contamination with dirt and faecal debris after flotation, making the microscopical detection easier. It was suggested that the oocysts remain concentrated in the lamina propria and that the sporocysts are released intermittently over time (Dubey et al., 2016; Moré et al., 2016).

It is known that dogs are DH for some *Sarcocystis* species affecting red deer such as *S. cervicanis* (Dubey et al., 2016). Therefore, and as DH for several *Sarcocystis* species affecting cervids are unknown, faecal samples of hunting dogs from Grisons were analysed. *Sarcocystis* sporocysts were detected in two of twelve analysed dogs. According to our questionnaire, both surveyed hunting dogs were regularly fed with raw meat or viscera form red deer and other hunted animals (Supplementary Table 3). In one of the dogs (Dog 5), molecular findings suggested a coinfection with *S. gracilis* and *S. capreolicanis*. These *Sarcocystis* species use roe deer as IH and dogs and foxes as DH (Dubey et al., 2016). In the further dog (Dog 6), cloned sequences with either 99.9% identity to GenBank sequences of *S. gracilis* (MN334289) or 98.8–99.1% identity with *S. linearis* (KY973371, MN334294) (Gjerde et al., 2017b; Rudaitytė-Lukošienė et al., 2020) and *S. taeniata* (KU753890) (Prakas et al., 2016) were obtained. These findings suggest that domestic dogs may be DH of *S. linearis* and/or *S. taeniata*.

## 5 Conclusion

This study revealed a high frequency of *Sarcocystis* infection in red deer in Grisons and the occurrence of at least five *Sarcocystis species* (i.e. S*. hjorti*, S*. venatoria*/*S. iberica, S. pilosa, S. linearis/S. taeniata*, and *S. ovalis*) in animals with grey-greenish tissue discolouration of the carcasses, and three species (i.e. S*. hjorti*, S*. venatoria*/*S. iberica* and *S. silva*) in animals with normal carcass appearance. First evidence of infection with *S. pilosa* and *S. silva* in red deer is provided; however, further morphological studies are needed to support these molecular findings.

Red foxes and hunting dogs from the region were shown to transmit *Sarcocystis* species affecting wild cervids, domestic ruminants and swine. Red foxes were confirmed as natural DH for *S. hjorti*, and hunting dogs are probably DH for *S. linearis/S. taeniata.* Moreover, also a *Sarcocystis* species transmitted by corvid birds (i.e. *S. ovalis*) was detected in red deer with eosinophilic myositis/fasciitis, representing the first record of this parasite in Central Europe.

## Authors contribution

WB and PD designed and supervised the study. WB supervised and performed laboratory work, analysed the results, and wrote the manuscript. CAAR carried out molecular analysis, was responsible for cloning work and molecular result analysis. DB organised and performed the sampling, carried out preliminary laboratory work and provided a draft of the study within the frame of his Master thesis at the Vetsuisse Faculty, University of Zurich. MR performed the histopathological analysis. All authors revised, contributed. and approved the manuscript.

## Declaration of competing interest

The authors declare that they have no known competing financial interests or personal relationships that could have appeared to influence the work reported in this paper.

## Figures and Tables

**figs1:**
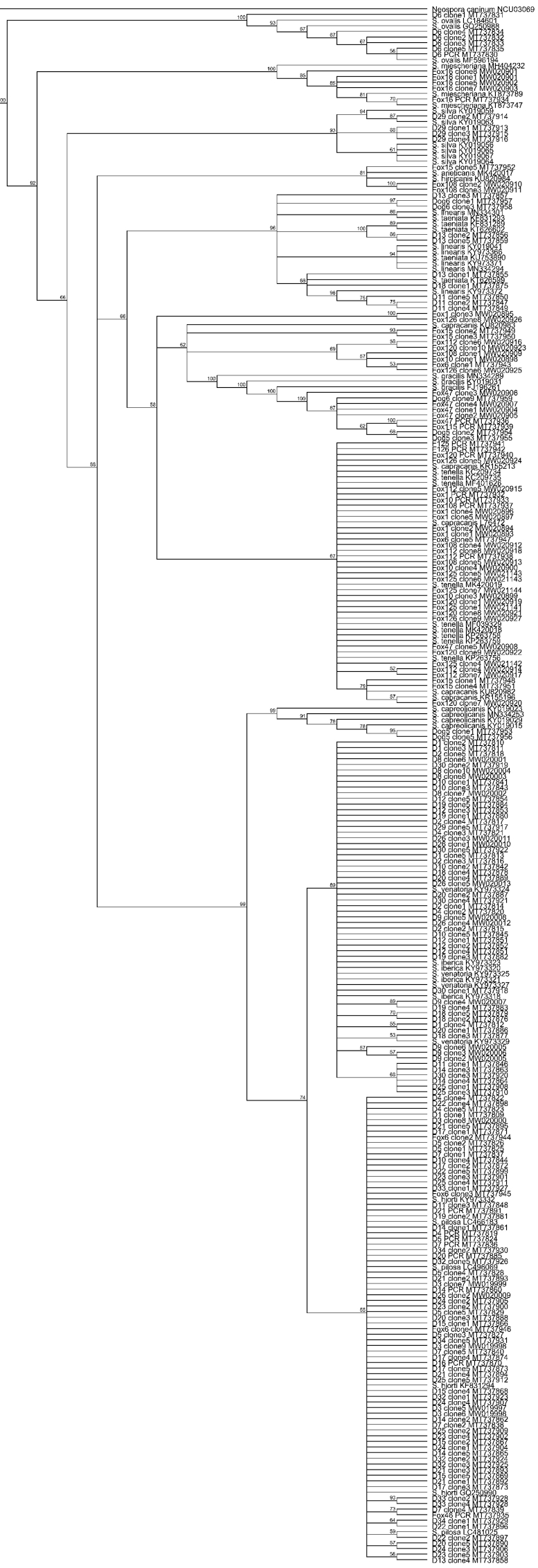
Phylogenetic tree for *Sarcocystis* species based on partial 18S rRNA sequences of 215 Taxa obtained in the present study and 61 GenBank® reference sequences according to Table 3, Table 4, inferred using the neighbour-joining method.
