# Supplementary material for: Sarcocystis infection in red deer (Cervus elaphus) with eosinophilic myositis/fasciitis in Switzerland and involvement of red foxes (Vulpesvulpes) and hunting dogs in the transmission
Source: Int J Parasitol Parasites Wildl. 2020 Oct 1;13:130–41. doi: 10.1016/j.ijppaw.2020.09.005 (PMC7551655; doi:10.1016/j.ijppaw.2020.09.005)
Supplement: Multimedia component 1 [file mmc1.docx]

**Supplementary Table 1**

Red Deer from Grisons, Switzerland, sampled for detection of *Sarcocystis* spp. infection: geographical coordinates of hunting sites and sampling date.

| **Red deer**  **No.** | **Hunting region** | **Geographical coordinates of the hunting sites^a)^** | **Sampling date** |
| --- | --- | --- | --- |
| 1 | **Davos** | 776 000/177 000 | 03.09.15 |
| 2 |  | 776 000/177 000 | 04.09.15 |
| 3 |  | 785 000/183 000 | 05.09.15 |
| 4 |  | 777 000/180 000 | 03.09.15 |
| 5 |  | 785 000/183 000 | 03.09.15 |
| 6 |  | 777 000/180 000 | 03.09.15 |
| 7 | **Rueun** | 727 163/184 197 | 04.09.15 |
| 8 |  | 745 749/182 714 | 03.09.15 |
| 9 |  | 721 490/178 916 | 26.09.15 |
| 10 | **Cunters** | 761 000/170 000 | 04.09.15 |
| 11 |  | 761 000/170 000 | 04.09.15 |
| 12 |  | 765 000/154 000 | 03.09.15 |
| 13 |  | 763 000/164 000 | 03.09.15 |
| 14 |  | 762 000/170 000 | 04.09.15 |
| 15 |  | 764 000/152 000 | 04.09.15 |
| 16 |  | 765 000/152 000 | 03.09.15 |
| 17 |  | 764 000/152 000 | 03.09.15 |
| 18 |  | 765 000/154 000 | 03.09.15 |
| 19 |  | 765 000/154 000 | 04.09.15 |
| 20 |  | 762 000/170 000 | 04.09.15 |
| 21 | **Filisur** | 772 574/171 841 | 19.06.15 |
| 22 |  | 772 000/171 000 | 20.06.15 |
| 23 |  | 772 000/171 000 | 19.06.15 |
| 24 |  | 772 990/169 280 | 20.06.15 |
| 25 |  | 774 000/171 000 | 13.06.15 |
| 26 |  | 765 000/169 000 | 13.06.15 |
| 27 | **Rueun** | 736 566/179 418 | 26.09.15 |
| 28 |  | 707 876/166 351 | 26.09.15 |
| 29 |  | 731 651/173 941 | 26.09.15 |
| 30 |  | 729 134/187 345 | 26.09.15 |
| 31 |  | 731 540/184 872 | 26.09.15 |
| 32 |  | 738 958/170 980 | 26.09.15 |
| 33 |  | 737 800/175 500 | 26.09.15 |
| 34 |  | 745 741/182 728 | 26.09.15 |

Supplementary Table 2

Red foxes (*Vulpes vulpes*) from Grisons, Switzerland, sampled for detection of *Sarcocystis* spp. infection in faeces (Fox 1-106) or intestine mucosa (Fox 107-126)

| **Fox**  **No.** | **Sampling**  **date** | **Age**  **U/J/A** | **Sex**  **U/M/F** | **Geographical coordinates of the sampling/hunting sites*** | **Microscopical detection of *Sarcocystis* sporocysts** | **Detection of *Sarcocystis***  **DNA** |
| --- | --- | --- | --- | --- | --- | --- |
| 1 | 30.11.2013 | U | U | 726601/167377 | Positive | Y |
| 2 | 24.12.2013 | U | U | 728.660/178359 | Positive | N |
| 3 | 24.12.2013 | U | U | 728.660/170.359 | Negative | n |
| 4 | 10.11.2013 | U | U | 725.949/166.909 | Negative | n |
| 5 | 24.12.2013 | U | U | 729.450/171.319 | Negative | n |
| 6 | 24.12.2013 | U | U | 728.660/170.359 | Positive | Y |
| 7 | 23.12.2013 | U | U | 730.876/173.832 | Negative | n |
| 8 | 23.12.2013 | U | U | 730.870/173.839 | Negative | n |
| 9 | 24.12.2013 | U | U | 728.660/170.359 | Negative | n |
| 10 | 24.12.2013 | U | U | 729.426/171.318 | Positive | Y |
| 11 | 04.12.2013 | U | U | 729020/171157 | Negative | n |
| 12 | 24.12.2013 | U | U | 728490/170166 | Positive | N |
| 13 | 08.12.2013 | U | U | 732450/163155 | Negative | n |
| 14 | 27.11.2013 | U | U | 733868/165669 | Negative | n |
| 15 | 27.11.2013 | U | U | 733870/166859 | Positive | Y |
| 16 | 07.01.2014 | U | U | 731650/163259 | Positive | Y |
| 17 | 06.12.2013 | U | U | 729350/160859 | Negative | n |
| 18 | 18.12.2013 | U | U | 732450/163159 | Negative | n |
| 19 | 02.12.2014 | A | M | 733870/166859 | Negative | n |
| 20 | 04.12.2013 | U | U | 725020/171159 | Negative | n |
| 21 | 21.01.2014 | A | M | 730843/180197 | Negative | n |
| 22 | 17.01.2014 | U | U | 730870/168327 | Negative | n |
| 23 | 07.01.2014 | J | W | 730843/180197 | Negative | n |
| \| 24 \| \| --- \| | 14.02.2014 | A | W | 726615/178286 | Negative | n |
| 25 | 21.12.2014 | J | M | 726615/178286 | Negative | n |
| 26 | 11.12.2014 | J | W | 726615/178286 | Negative | n |
| 27 | 02.01.2014 | A | M | 726675/178733 | Negative | n |
| 28 | 15.02.2014 | A | M | 726615/178280 | Negative | n |
| 29 | 07.02.2014 | U | U | 738487/184693 | Negative | n |
| 30 | 04.01.2014 | U | U | 736281/183628 | Negative | n |
| 31 | 20.01.2014 | U | u | 739292/183219 | Negative | n |
| 32 | 13.09.2013 | U | U | 742944/180668 | Negative | n |
| 33 | 06.09.2013 | U | U | 738612/185929 | Positive | N |
| 34 | 15.01.2014 | U | U | 741795/183111 | Negative | n |
| 35 | 13.12.2013 | U | U | 742955/183877 | Negative | n |
| 36 | 06.02.2013 | U | U | 742955/183877 | Negative | n |
| 37 | 15.12.2014 | U | U | 742955/183877 | Negative | n |
| 38 | 12.12.2014 | J | M | 737714/182242 | Negative | n |
| 39 | 06.12.2014 | J | M | 736458/181340 | Negative | n |
| 40 | 08.12.2014 | A | W | 736458/181340 | Negative | n |
| 41 | 18.01.2014 | A | M | 736145/185087 | Negative | n |
| 42 | 26.01.2015 | A | M | 733308/173502 | Negative | n |
| 43 | 26.01.2015 | A | W | 733163/173802 | Negative | n |
| 44 | 26.01.2015 | A | M | 733163/173802 | Negative | n |
| 45 | 26.01.2015 | A | M | 733308/173502 | Negative | n |
| 46 | 26.01.2015 | A | M | 733163/173502 | Positive | Y |
| 47 | 26.01.2015 | A | W | 733163/173502 | Positive | Y |
| 48 | 26.01.2015 | J | M | 733163/173882 | Negative | n |
| 49 | 26.01.2015 | A | M | 733163/173882 | Negative | n |
| 50 | 22.02.2014 | J | M | 736145/185087 | Negative | n |
| 51 | 02.12.2014 | A | W | 736145/185087 | Negative | n |
| 52 | 14.12.2014 | A | M | 736145/185087 | Negative | n |
| 53 | 23.02.2014 | A | M | 736145/185087 | Negative | n |
| 54 | 02.02.2015 | A | M | 726394/178645 | Negative | n |
| 55 | 04.02.2015 | A | W | 726394/178645 | Negative | n |
| 56 | 23.01.2015 | J | W | 735706/179880 | Negative | n |
| 57 | 26.01.2015 | J | W | 735706/179880 | Negative | n |
| 58 | 26.01.2015 | J | W | 735706/179880 | Negative | n |
| 59 | 27.01.2015 | A | W | 728386/181212 | Negative | n |
| 60 | 17.01.2015 | A | M | 735706/179880 | Negative | n |
| 61 | 09.03.2014 | U | U | 736504/176447 | Negative | n |
| 62 | 02.02.2014 | U | U | 735243/176798 | Negative | n |
| 63 | 15.01.2014 | U | U | 735243/176798 | Negative | n |
| 64 | 10.12.2014 | U | U | 736542/182437 | Negative | n |
| 65 | 15.12.2014 | U | U | 736627/182437 | Negative | n |
| 66 | 14.02.2014 | U | U | 730643/182501 | Negative | n |
| 67 | 01.09.2015 | A | M | 795426/131918 | Negative | n |
| 68 | 09.12.2014 | U | U | 732627/182420 | Negative | n |
| 69 | 25.01.2014 | A | M | 726615/178288 | Negative | n |
| 70 | 08.02.2014 | A | W | 726615/178288 | Negative | n |
| 71 | 27.02.2014 | A | W | 723487/178730 | Negative | n |
| 72 | 11.12.2013 | A | W | 726615/178288 | Negative | n |
| 73 | 01.01.2014 | J | M | 723487/178730 | Negative | n |
| 74 | 27.02.2014 | J | W | 723487/178730 | Negative | n |
| 75 | 24.05.2015 | J | W | 769050/152617 | Negative | n |
| 76 | 30.05.2015 | J | M | 765760/166509 | Negative | n |
| 77 | 23.05.2015 | J | M | 761499/162956 | Negative | n |
| 78 | 06.07.2015 | J | W | 769513/143689 | Negative | n |
| 79 | 08.04.2015 | A | M | 802718/133187 | Negative | n |
| 80 | 17.06.2015 | J | W | 770200/148228 | Negative | n |
| 81 | 15.09.2015 | J | M | 766600/157377 | Negative | n |
| 82 | 14.09.2015 | J | M | 766660/150357 | Negative | n |
| 83 | 20.09.2015 | J | W | 768660/162368 | Negative | n |
| 84 | 15.09.2015 | U | U | 765949/166909 | Negative | n |
| 85 | 15.09.2015 | U | U | 769458/171318 | Negative | n |
| 86 | 14.09.2015 | U | U | 768660/170358 | Negative | n |
| 87 | 15.09.2015 | U | U | 760876/173831 | Negative | n |
| 88 | 22.09.2015 | U | U | 796087/133898 | Negative | n |
| 89 | 01.10.2015 | U | U | 798560/130344 | Negative | n |
| 90 | 01.10.2015 | U | U | 797721/132/767 | Negative | n |
| 91 | 03.10.2015 | J | M | 745121/182638 | Negative | n |
| 92 | 03.10.2015 | J | M | 745121/182638 | Negative | n |
| 93 | 03.10.2015 | J | M | 740460/183527 | Negative | n |
| 94 | 03.10.2015 | J | M | 740460/183527 | Negative | n |
| 95 | 03.10.2015 | U | U | 745350/180324 | Negative | n |
| 96 | 24.12.2015 | U | U | 723487/178738 | Negative | n |
| 97 | 24.12.2014 | U | U | 726601/167372 | Negative | n |
| 98 | 24.12.2014 | U | U | 725650/167224 | Positive | N |
| 99 | 24.12.2014 | U | U | 729660/170351 | Negative | n |
| 100 | 24.12.2014 | U | U | 729660/170351 | Negative | n |
| 101 | 03.10.2015 | U | U | 726188/167112 | Negative | n |
| 102 | 01.10.2015 | U | U | 723487/178735 | Negative | n |
| 103 | 01.10.2015 | U | U | 727889/158789 | Negative | n |
| 104 | 26.09.2015 | U | U | 728490/170168 | Negative | n |
| 105 | 03.10.2015 | U | U | 726600/167379 | Negative | n |
| 106 | 26.09.2015 | U | U | 728490/170168 | Negative | n |
| 107 | 03.03.2015 | A | M | 785.026/153.522 | Negative | n |
| 108 | 08.04.2015 | A | W | 802718/133182 | Positive | Y |
| 109 | 19.05.2015 | J | M | 771541/148082 | Negative | n |
| 110 | 26.06.2015 | J | M | 769814/144/746 | Negative | n |
| 111 | 29.10.2015 | J | W | 760576/147872 | Negative | n |
| 112 | 04.12.2016 | J | M | 766027/169573 | Positive | Y |
| 113 | 18.12.2015 | J | M | 771551/170368 | Negative | n |
| 114 | 19.12.2015 | J | M | 775135/170527 | Negative | n |
| 115 | 08.01.2016 | J | M | 771956/172066 | Positive | Y |
| 116 | 09.01.2016 | A | M | 771000/171000 | Negative | n |
| 117 | 09.01.2016 | A | W | 771000/171000 | Negative | n |
| 118 | 12.01.2016 | A | W | 772165/170277 | Negative | n |
| 119 | 16.01.2016 | A | W | 774606/170642 | Negative | n |
| 120 | 16.01.2016 | J | M | 764000/164000 | Positive | Y |
| 121 | 19.01.2016 | A | M | 765646/163838 | Negative | n |
| 122 | 21.01.2016 | J | W | 764870/163462 | Negative | n |
| 123 | 23.01.2016 | J | W | 765000/164000 | Negative | n |
| 124 | 23.01.2016 | J | M | 781000/185000 | Negative | n |
| 125 | 23.01.2016 | A | M | 782000/186000 | Positive | Y |
| 126 | 30.01.2016 | A | M | 782000/186000 | Positive | Y |

U: Unknown; J: Juvenile; A: Adult; F: female; M: male; Y: yes; N: no; n: not analysed

*Swiss coordinate system «Swiss Grid» 1903

Supplementary Table 3

Hunting dogs from Grisons, Switzerland, sampled for detection of *Sarcocystis* spp. in faeces

| **Dog No** | **Age (Years)** | **Sex**  **(F/M)** | **Breed** | **Fed with raw meat or**  **viscera from red deer and other hunted animals** | **Main hunting region** | **Microscopical detection of *Sarcocystis*** | **Detection of *Sarcocystis***  **DNA** |
| --- | --- | --- | --- | --- | --- | --- | --- |
| 1 | 5 | M | Black Forest Hound | Y | Val Müstair | N | n |
| 2 | 8.5 | M | Bavarian Mountain Hound | Y | Vals | N | n |
| 3 | 1.5 | M | Black Forest Hound | Y | Val Poschiavo | N | n |
| 4 | 3.5 | F | Labrador Retriever | Y | St. Moritz, Celerina, Samedan, Pontresina | N | n |
| 5 | 0.75 | F | Hanover Hound | Y | Canton Grisons | Y | Y |
| 6 | 13 | M | Magyar Viszla | Y | Canton Grisons | Y | Y |
| 7 | 5 | F | Hanover Hound | Y | Zernez/Val Müstair, Samnaun, | N | n |
| 8 | 0.75 | M | Hanover Hound | Y | Zernez/Val Müstair, Samnaun, | N | n |
| 9 | 6 | M | Swiss hound | Y | Zernez/Val Müstair, Samnaun, | N | n |
| 10 | 3 | M | U | Y | Canton Grisons | N | n |
| 11 | 4.5 | F | U | Y | Canton Grisons | N | n |
| 12 | 2 | M | U | Y | Canton Grisons | N | n |

F: female; M: male; U: not informed; Y: yes; N: no; n: not analysed

**Supplementary Table 4**: Description of *Sarcocystis* spp. 18S rRNA gene sequences amplified from skeletal muscle of red deer from Grisons, Switzerland, obtained by direct sequencing of the PCR products or after cloning into vector plasmids

| Animal  ID | Sequence  ID | Sequence  length (bp) | BLASTn identity | (%) | GenBank®  accession no.  (reference sequence) | Reference | *Sarcocystis* sp.  (this study) | GenBank®  accession no.  (this study) |
| --- | --- | --- | --- | --- | --- | --- | --- | --- |
| Deer 1 | D1 clone1 | 652 | *S. hjorti* | 99.7 | KY973332 | (Gjerde et al. 2017b) | *S. hjorti* | MT737809 |
|  | D1 clone2 | 664 | *S. venatoria* | 99.9 | KY973325 | (Gjerde et al. 2017b) | *Sarcocystis* sp. | MT737810 |
|  |  |  | *S. iberica* | 99.6 | KY973318 | (Gjerde et al. 2017b) |  |  |
|  | D1 clone3 | 666 | *S. venatoria* | 99.6 | KY973327 | (Gjerde et al. 2017b) | *Sarcocystis* sp. | MT737811 |
|  |  |  | *S. iberica* | 99.6 | KY973321 | (Gjerde et al. 2017b) |  |  |
|  | D1 clone4 | 662 | *S. venatoria* | 99.1 | KY973324 | (Gjerde et al. 2017b) | *Sarcocystis* sp. | MT737812 |
|  |  |  | *S. iberica* | 98.9 | KY973318 | (Gjerde et al. 2017b) |  |  |
|  | D1 clone5 | 662 | *S. venatoria* | 99.6 | KY973324 | (Gjerde et al. 2017b) | *Sarcocystis* sp. | MT737813 |
|  |  |  | *S. iberica* | 99.7 | KY973318 | (Gjerde et al. 2017b) |  |  |
| Deer 2 | D2 clone1 | 666 | *S. venatoria* | 99.7 | KY973327 | (Gjerde et al. 2017b) | *Sarcocystis* sp. | MT737814 |
|  |  |  | *S. iberica* | 99.6 | KY973321 | (Gjerde et al. 2017b) |  |  |
|  | D2 clone2 | 664 | *S. venatoria* | 99.6 | KY973325 | (Gjerde et al. 2017b) | *Sarcocystis* sp. | MT737815 |
|  |  |  | *S. iberica* | 99.3 | KY973318 | (Gjerde et al. 2017b) |  |  |
|  | D2 clone3 | 662 | *S. venatoria* | 99.9 | KY973324 | (Gjerde et al. 2017b) | *Sarcocystis* sp. | MT737816 |
|  |  |  | *S. iberica* | 99.7 | KY973318 | (Gjerde et al. 2017b) |  |  |
|  | D2 clone4 | 662 | *S. venatoria* | 99.7 | KY973324 | (Gjerde et al. 2017b) | *Sarcocystis* sp. | MT737817 |
|  |  |  | *S. iberica* | 99.6 | KY973318 | (Gjerde et al. 2017b) |  |  |
|  | D2 clone5 | 666 | *S. venatoria* | 99.1 | KY973327 | (Gjerde et al. 2017b) | *Sarcocystis* sp. | MT737818 |
|  |  |  | *S. iberica* | 99.1 | KY973321 | (Gjerde et al. 2017b) |  |  |
| Deer 3 | D3 clone5 | 654 | *S. hjorti* | 99.9 | GQ250990 | (Dahlgren and Gjerde 2010a) | *S. hjorti* | MW019997 |
|  | D3 clone6 | 654 | *S. hjorti* | 100 | GQ250990 | (Dahlgren and Gjerde 2010a) | *S. hjorti* | MW019998 |
|  | D3 clone7 | 654 | *S. hjorti* | 99.9 | GQ250990 | (Dahlgren and Gjerde 2010a) | *S. hjorti* | MW019999 |
|  | D3 clone8 | 654 | *S. hjorti* | 99.7 | GQ250990 | (Dahlgren and Gjerde 2010a) | *S. hjorti* | MW020000 |
|  | D3 clone9 | 654 | *S. hjorti* | 100 | GQ250990 | (Dahlgren and Gjerde 2010a) | *S. hjorti* | MW019998 |
| Deer 4 | D4 PCR | 654 | *S. hjorti* | 100 | GQ250990 | (Dahlgren and Gjerde 2010a) | *S. hjorti* | MT737819 |
|  | D4 clone2 | 666 | *S. venatoria* | 99.7 | KY973327 | (Gjerde et al. 2017b) | *Sarcocystis* sp. | MT737820 |
|  |  |  | *S. iberica* | 99.7 | KY973321 | (Gjerde et al. 2017b) |  |  |
|  | D4 clone3 | 666 | *S. venatoria* | 99.3 | KY973327 | (Gjerde et al. 2017b) | *Sarcocystis* sp. | MT737821 |
|  |  |  | *S. iberica* | 99.3 | KY973321 | (Gjerde et al. 2017b) |  |  |
|  | D4 clone4 | 652 | *S. hjorti* | 99.5 | KY973332 | (Gjerde et al. 2017b) | *S. hjorti* | MT737822 |
|  | D4 clone5 | 654 | *S. hjorti* | 99.7 | KF831294 | (Gjerde 2014a) | *S. hjorti* | MT737823 |
| Deer 5 | D5 PCR | 630 | *S. hjorti* | 100 | GQ250990 | (Dahlgren and Gjerde 2010a) | *S. hjorti* | MT737824 |
|  | D5 clone1 | 652 | *S. hjorti* | 100 | KY973332 | (Gjerde et al. 2017b) | *S. hjorti* | MT737825 |
|  | D5 clone2 | 652 | *S. hjorti* | 99.7 | KY973332 | (Gjerde et al. 2017b) | *S. hjorti* | MT737826 |
|  | D5 clone3 | 654 | *S. hjorti* | 99.7 | KF831294 | (Gjerde 2014a) | *S. hjorti* | MT737827 |
|  | D5 clone4 | 654 | *S. hjorti* | 98.9 | KF831294 | (Gjerde 2014a) | *S. hjorti* | MT737828 |
|  | D5 clone5 | 654 | *S. hjorti* | 99.9 | KF831294 | (Gjerde 2014a) | *S. hjorti* | MT737829 |
| Deer 6 | D6 PCR | 630 | *S. ovalis* | 100 | GQ250988 | (Dahlgren and Gjerde 2010a) | *S. ovalis* | MT737830 |
|  | D6 clone1 | 652 | *S. ovalis* | 99.7 | LC184601 | (Irie et al., 2017) | *S. ovalis* | MT737831 |
|  | D6 clone2 | 654 | *S. ovalis* | 99.9 | LC184601 | (Irie et al., 2017) | *S. ovalis* | MT737832 |
|  | D6 clone3 | 654 | *S. ovalis* | 99.9 | LC184601 | (Irie et al., 2017) | *S. ovalis* | MT737833 |
|  | D6 clone4 | 654 | *S. ovalis* | 100 | LC184601 | (Irie et al., 2017) | *S. ovalis* | MT737834 |
|  | D6 clone5 | 654 | *S. ovalis* | 99.2 | LC184601 | (Irie et al., 2017) | *S. ovalis* | MT737835 |
| Deer 7 | D7 PCR | 628 | *S. hjorti* | 100 | GQ250990 | (Dahlgren and Gjerde 2010a) | *S. hjorti* | MT737836 |
|  | D7 clone1 | 652 | *S. hjorti* | 100 | KY973332 | (Gjerde et al. 2017b) | *S. hjorti* | MT737837 |
|  | D7 clone2 | 654 | *S. hjorti* | 99.9 | KF831294 | (Gjerde 2014a) | *S. hjorti* | MT737838 |
|  | D7 clone4 | 654 | *S. hjorti* | 99.2 | KF831294 | (Gjerde 2014a) | *S. hjorti* | MT737839 |
|  | D7 clone5 | 654 | *S. hjorti* | 99.9 | KF831294 | (Gjerde 2014a) | *S. hjorti* | MT737840 |
| Deer 8 | D8 clone6 | 662 | *S. venatoria*  *S. iberica* | 99.6  99.4 | KY973324  KY973318 | (Gjerde et al. 2017b)  (Gjerde et al. 2017b) | *Sarcocystis* sp. | MW020001 |
|  | D8 clone7 | 666 | *S. venatoria*  *S. iberica* | 99.4  99.4 | KY973327  KY973321 | (Gjerde et al. 2017b)  (Gjerde et al. 2017b) | *Sarcocystis* sp. | MW020002 |
|  | D8 clone8 | 664 | *S. venatoria*  *S. iberica* | 99.9  99.9 | KY973327  KY973321 | (Gjerde et al. 2017b)  (Gjerde et al. 2017b) | *Sarcocystis* sp. | MW020003 |
|  | D8 clone10 | 662 | *S. venatoria*  *S. iberica* | 99.7  99.6 | KY973324  KY973321 | (Gjerde et al. 2017b)  (Gjerde et al. 2017b) | *Sarcocystis* sp. | MW020004 |
| Deer 9 | D9 clone2 | 664 | *S. venatoria*  *S. iberica* | 99.7  99.6 | KY973325  KY973318 | (Gjerde et al. 2017b)  (Gjerde et al. 2017b) | *Sarcocystis* sp. | MW020005 |
|  | D9 clone3 | 664 | *S. venatoria*  *S. iberica* | 99.6  99.3 | KY973325  KY973318 | (Gjerde et al. 2017b)  (Gjerde et al. 2017b) | *Sarcocystis* sp. | MW020006 |
|  | D9 clone4 | 666 | *S. venatoria*  *S. iberica* | 99.7  99.7 | KY973327  KY973323 | (Gjerde et al. 2017b)  (Gjerde et al. 2017b) | *Sarcocystis* sp. | MW020007 |
|  | D9 clone5 | 666 | *S. venatoria*  *S. iberica* | 99.6  99.6 | KY973327  KY973321 | (Gjerde et al. 2017b)  (Gjerde et al. 2017b) | *Sarcocystis* sp. | MW020008 |
|  | D9 clone6 | 664 | *S. venatoria*  *S. iberica* | 99.7  99.4 | KY973325  KY973318 | (Gjerde et al. 2017b)  (Gjerde et al. 2017b) | *Sarcocystis* sp. | MW020005 |
| Deer 10 | D10 clone1 | 662 | *S. venatoria* | 99.9 | KY973324 | (Gjerde et al. 2017b) | *Sarcocystis* sp. | MT737841 |
|  |  |  | *S. iberica* | 99.7 | KY973318 | (Gjerde et al. 2017b) |  |  |
|  | D10 clone2 | 662 | *S. venatoria* | 99.9 | KY973324 | (Gjerde et al. 2017b) | *Sarcocystis* sp. | MT737842 |
|  |  |  | *S. iberica* | 99.7 | KY973318 | (Gjerde et al. 2017b) |  |  |
|  | D10 clone3 | 662 | *S. venatoria* | 99.6 | KY973324 | (Gjerde et al. 2017b) | *Sarcocystis* sp. | MT737843 |
|  |  |  | *S. iberica* | 99.4 | KY973318 | (Gjerde et al. 2017b) |  |  |
|  | D10 clone4 | 652 | *S. hjorti* | 100 | KY973332 | (Gjerde et al. 2017b) | *S. hjorti* | MT737844 |
|  | D10 clone5 | 664 | *S. venatoria* | 99.7 | KY973325 | (Gjerde et al. 2017b) | *Sarcocystis* sp. | MT737845 |
|  |  |  | *S. iberica* | 99.4 | KY973318 | (Gjerde et al. 2017b) |  |  |
| Deer 11 | D11 clone1 | 664 | *S. venatoria* | 99.4 | KY973325 | (Gjerde et al. 2017b) | *Sarcocystis* sp. | MT737846 |
|  |  |  | *S. iberica* | 99.1 | KY973318 | (Gjerde et al. 2017b) |  |  |
|  | D11 clone2 | 660 | *S. linearis* | 99.9 | KY973372 | (Gjerde et al. 2017b) | *Sarcocystis* sp. | MT737847 |
|  |  |  | *S. taeniata* | 99.4 | KU753890 | (Prakas et al. 2016) |  |  |
|  | D11 clone3 | 654 | *S. hjorti* | 99.4 | KF831294 | (Gjerde 2014a) | *S. hjorti* | MT737848 |
|  | D11 clone4 | 660 | *S. linearis* | 99.9 | KY973372 | (Gjerde et al. 2017b) | *Sarcocystis* sp. | MT737849 |
|  |  |  | *S. taeniata* | 99.4 | KU753890 | (Prakas et al. 2016) |  |  |
|  | D11 clone5 | 660 | *S. linearis* | 100 | KY973372 | (Gjerde et al. 2017b) | *Sarcocystis* sp. | MT737850 |
|  |  |  | *S. taeniata* | 99.6 | KU753890 | (Prakas et al. 2016) |  |  |
| Deer 12 | D12 clone1 | 664 | *S. venatoria* | 99.9 | KY973325 | (Gjerde et al. 2017b) | *Sarcocystis* sp. | MT737851 |
|  |  |  | *S. iberica* | 99.6 | KY973318 | (Gjerde et al. 2017b) |  |  |
|  | D12 clone2 | 664 | *S. venatoria* | 99.7 | KY973325 | (Gjerde et al. 2017b) | *Sarcocystis* sp. | MT737852 |
|  |  |  | *S. iberica* | 99.4 | KY973318 | (Gjerde et al. 2017b) |  |  |
|  | D12 clone3 | 664 | *S. venatoria* | 99.7 | KY973325 | (Gjerde et al. 2017b) | *Sarcocystis* sp. | MT737853 |
|  |  |  | *S. iberica* | 99.4 | KY973318 | (Gjerde et al. 2017b) |  |  |
|  | D12 clone4 | 664 | *S. venatoria* | 99.9 | KY973325 | (Gjerde et al. 2017b) | *Sarcocystis* sp. | MT737851 |
|  |  |  | *S. iberica* | 99.6 | KY973318 | (Gjerde et al. 2017b) |  |  |
|  | D12 clone5 | 664 | *S. venatoria* | 99.7 | KY973325 | (Gjerde et al. 2017b) | *Sarcocystis* sp. | MT737854 |
|  |  |  | *S. iberica* | 99.4 | KY973318 | (Gjerde et al. 2017b) |  |  |
| Deer 13 | D13 clone1 | 660 | *S. linearis* | 99.9 | KY973371 | (Gjerde et al. 2017b) | *Sarcocystis* sp. | MT737855 |
|  |  |  | *S. taeniata* | 99.7 | KU753890 | (Prakas et al. 2016) |  |  |
|  | D13 clone2 | 658 | *S. taeniata* | 99.9 | KT626602 | (Reissig et al. 2016) | *Sarcocystis* sp. | MT737856 |
|  |  |  | *S. linearis* | 98.2 | KY973372 | (Gjerde et al. 2017b) |  |  |
|  | D13 clone3 | 650 | *S. linearis* | 98.9 | MN334301 | (Rudaitytė-Lukošienė et al. 2020) | *Sarcocystis* sp. | MT737857 |
|  |  |  | *S. taeniata* | 98.8 | KF831293 | (Gjerde 2014a) |  |  |
|  | D13 clone4 | 654 | *S. hjorti* | 99.2 | KF831294 | (Gjerde 2014a) | *S. hjorti* | MT737858 |
|  | D13 clone5 | 658 | *S. taeniata* | 99.5 | KT626602 | (Reissig et al. 2016) | *Sarcocystis* sp. | MT737859 |
|  |  |  | *S. linearis* | 97.9 | KY973372 | (Gjerde et al. 2017b) |  |  |
| Deer 14 | D14PCR | 529 | *S. pilosa* | 100 | LC466183 | (Irie et al. 2019) | *S. pilosa* | MT737860 |
|  | D14 clone1 | 653 | *S. pilosa* | 99.7 | LC466183 | (Irie et al. 2019) | *S. pilosa* | MT737861 |
|  | D14 clone2 | 654 | *S. hjorti* | 98.9 | KF831294 | (Gjerde 2014a) | *S. hjorti* | MT737862 |
|  | D14 clone3 | 664 | *S. venatoria* | 98.9 | KY973325 | (Gjerde et al. 2017b) | *Sarcocystis* sp*.* | MT737863 |
|  |  |  | *S. iberica* | 98.6 | KY973318 | (Gjerde et al. 2017b) |  |  |
|  | D14 clone4 | 664 | *S. venatoria* | 99.4 | KY973325 | (Gjerde et al. 2017b) | *Sarcocystis* sp*.* | MT737864 |
|  |  |  | *S. iberica* | 99.1 | KY973318 | (Gjerde et al. 2017b) |  |  |
|  | D14 clone5 | 654 | *S. hjorti* | 99.9 | KF831294 | (Gjerde 2014a) | *S. hjorti* | MT737865 |
| Deer 15 | D15 clone1 | 654 | *S. hjorti* | 99.9 | KF831294 | (Gjerde 2014a) | *S. hjorti* | MT737866 |
|  | D15 clone2 | 654 | *S. hjorti* | 100 | KF831294 | (Gjerde 2014a) | *S. hjorti* | MT737867 |
|  | D15 clone4 | 654 | *S. hjorti* | 99.7 | KF831294 | (Gjerde 2014a) | *S. hjorti* | MT737868 |
|  | D15 clone5 | 654 | *S. hjorti* | 99.7 | KF831294 | (Gjerde 2014a) | *S. hjorti* | MT737869 |
| Deer 16 | D16 PCR | 654 | *S. hjorti* | 100 | GQ250990 | (Dahlgren and Gjerde 2010a) | *S. hjorti* | MT737870 |
| Deer 17 | D17 clone1 | 652 | *S. hjorti* | 99.9 | KY973332 | (Gjerde et al. 2017b) | *S. hjorti* | MT737871 |
|  | D17 clone2 | 652 | *S. hjorti* | 99.7 | KY973332 | (Gjerde et al. 2017b) | *S. hjorti* | MT737872 |
|  | D17 clone3 | 654 | *S. hjorti* | 100 | KF831294 | (Gjerde 2014a) | *S. hjorti* | MT737873 |
|  | D17 clone4 | 654 | *S. hjorti* | 99.9 | KF831294 | (Gjerde 2014a) | *S. hjorti* | MT737874 |
|  | D17 clone5 | 654 | *S. hjorti* | 100 | KF831294 | (Gjerde 2014a) | *S. hjorti* | MT737873 |
| Deer 18 | D18 clone1 | 660 | *S. linearis* | 99.2 | KY973372 | (Gjerde et al. 2017b) | *Sarcocystis* sp*.* | MT737875 |
|  |  |  | *S. taeniata* | 98.8 | KU753890 | (Prakas et al. 2016) |  |  |
|  | D18 clone2 | 666 | *S. venatoria* | 99.6 | KY973327 | (Gjerde et al. 2017b) | *Sarcocystis* sp*.* | MT737876 |
|  |  |  | *S. iberica* | 99.6 | KY973321 | (Gjerde et al. 2017b) |  |  |
|  | D18 clone3 | 662 | *S. venatoria* | 99.7 | KY973324 | (Gjerde et al. 2017b) | *Sarcocystis* sp*.* | MT737877 |
|  |  |  | *S. iberica* | 99.6 | KY973318 | (Gjerde et al. 2017b) |  |  |
|  | D18 clone4 | 662 | *S. venatoria* | 100 | KY973324 | (Gjerde et al. 2017b) | *Sarcocystis* sp*.* | MT737878 |
|  |  |  | *S. iberica* | 99.9 | KY973318 | (Gjerde et al. 2017b) |  |  |
|  | D18 clone5 | 662 | *S. venatoria* | 99.7 | KY973324 | (Gjerde et al. 2017b) | *Sarcocystis* sp*.* | MT737879 |
|  |  |  | *S. iberica* | 99.5 | KY973318 | (Gjerde et al. 2017b) |  |  |
| Deer 19 | D19 clone1 | 664 | *S. venatoria* | 99.7 | KY973325 | (Gjerde et al. 2017b) | *Sarcocystis* sp*.* | MT737880 |
|  |  |  | *S. iberica* | 99.4 | KY973318 | (Gjerde et al. 2017b) |  |  |
|  | D19 clone2 | 653 | *S. pilosa* | 99.9 | LC466183 | (Irie et al. 2019) | *S. pilosa* | MT737881 |
|  | D19 clone3 | 664 | *S. venatoria* | 99.9 | KY973325 | (Gjerde et al. 2017b) | *Sarcocystis* sp*.* | MT737882 |
|  |  |  | *S. iberica* | 99.6 | KY973318 | (Gjerde et al. 2017b) |  |  |
|  | D19 clone4 | 666 | *S. venatoria* | 99.4 | KY973327 | (Gjerde et al. 2017b) | *Sarcocystis* sp*.* | MT737883 |
|  |  |  | *S. iberica* | 99.4 | KY973323 | (Gjerde et al. 2017b) |  |  |
|  | D19 clone5 | 664 | *S. venatoria* | 99.6 | KY973325 | (Gjerde et al. 2017b) | *Sarcocystis* sp*.* | MT737884 |
|  |  |  | *S. iberica* | 99.3 | KY973318 | (Gjerde et al. 2017b) |  |  |
| Deer 20 | D20PCR | 618 | *S. hjorti* | 100 | GQ250990 | (Dahlgren and Gjerde 2010a) | *S. hjorti* | MT737885 |
|  | D20 clone1 | 662 | *S. venatoria* | 99.7 | KY973324 | (Gjerde et al. 2017b) | *Sarcocystis* sp*.* | MT737886 |
|  |  |  | *S. iberica* | 99.6 | KY973318 | (Gjerde et al. 2017b) |  |  |
|  | D20 clone2 | 664 | *S. venatoria* | 99.7 | KY973325 | (Gjerde et al. 2017b) | *Sarcocystis* sp*.* | MT737887 |
|  |  |  | *S. iberica* | 99.4 | KY973318 | (Gjerde et al. 2017b) |  |  |
|  | D20 clone3 | 654 | *S. hjorti* | 100 | KF831294 | (Gjerde 2014a) | *S. hjorti* | MT737888 |
|  | D20 clone4 | 662 | *S. venatoria* | 100 | KY973324 | (Gjerde et al. 2017b) | *Sarcocystis* sp*.* | MT737889 |
|  |  |  | *S. iberica* | 99.9 | KY973318 | (Gjerde et al. 2017b) |  |  |
|  | D20 clone5 | 652 | *S. hjorti* | 99.7 | KY973332 | (Gjerde et al. 2017b) | *S. hjorti* | MT737890 |
| Deer 21 | D21PCR | 636 | *S. hjorti* | 100 | GQ250990 | (Dahlgren and Gjerde 2010a) | *S. hjorti* | MT737891 |
|  | D21 clone1 | 654 | *S. hjorti* | 99.9 | KF831294 | (Gjerde 2014a) | *S. hjorti* | MT737892 |
|  | D21 clone2 | 654 | *S. hjorti* | 100 | KF831294 | (Gjerde 2014a) | *S. hjorti* | MT737893 |
|  | D21 clone3 | 654 | *S. hjorti* | 100 | KF831294 | (Gjerde 2014a) | *S. hjorti* | MT737893 |
|  | D21 clone4 | 654 | *S. hjorti* | 99.9 | KF831294 | (Gjerde 2014a) | *S. hjorti* | MT737894 |
|  | D21 clone5 | 652 | *S. hjorti* | 99.5 | KY973332 | (Gjerde et al. 2017b) | *S. hjorti* | MT737895 |
| Deer 22 | D22 clone1 | 652 | *S. hjorti* | 99.5 | KY973332 | (Gjerde et al. 2017b) | *S. hjorti* | MT737896 |
|  | D22 clone2 | 654 | *S. hjorti* | 99.9 | KF831294 | (Gjerde 2014a) | *S. hjorti* | MT737897 |
|  | D22 clone4 | 651 | *S. hjorti* | 99.5 | KY973332 | (Gjerde et al. 2017b) | *S. hjorti* | MT737898 |
|  | D22 clone5 | 652 | *S. hjorti* | 100 | KY973332 | (Gjerde et al. 2017b) | *S. hjorti* | MT737899 |
| Deer 23 | D23 clone2 | 654 | *S. hjorti* | 99.7 | KF831294 | (Gjerde et al. 2017b) | *S. hjorti* | MT737900 |
|  | D23 clone3 | 652 | *S. hjorti* | 99.9 | KY973332 | (Gjerde et al. 2017b) | *S. hjorti* | MT737901 |
|  | D23 clone4 | 654 | *S. hjorti* | 99.9 | KF831294 | (Gjerde 2014a) | *S. hjorti* | MT737902 |
|  | D23 clone5 | 654 | *S. hjorti* | 99.5 | KF831294 | (Gjerde 2014a) | *S. hjorti* | MT737903 |
| Deer 24 | D24 clone1 | 654 | *S. hjorti* | 100 | KF831294 | (Gjerde 2014a) | *S. hjorti* | MT737904 |
|  | D24 clone2 | 654 | *S. hjorti* | 99.9 | KF831294 | (Gjerde 2014a) | *S. hjorti* | MT737905 |
|  | D24 clone3 | 652 | *S. hjorti* | 99.9 | KY973332 | (Gjerde et al. 2017b) | *S. hjorti* | MT737906 |
|  | D24 clone4 | 654 | *S. hjorti* | 99.9 | KF831294 | (Gjerde 2014a) | *S. hjorti* | MT737907 |
| Deer 25 | D25 clone1 | 664 | *S. venatoria* | 99.7 | KY973325 | (Gjerde et al. 2017b) | *Sarcocystis* sp*.* | MT737908 |
|  |  |  | *S. iberica* | 99.4 | KY973318 | (Gjerde et al. 2017b) |  |  |
|  | D25 clone2 | 654 | *S. hjorti* | 99.4 | KF831294 | (Gjerde 2014a) | *S. hjorti* | MT737909 |
|  | D25 clone3 | 664 | *S. venatoria* | 99.6 | KY973325 | (Gjerde et al. 2017b) | *Sarcocystis* sp*.* | MT737910 |
|  |  |  | *S. iberica* | 99.3 | KY973318 | (Gjerde et al. 2017b) |  |  |
|  | D25 clone4 | 652 | *S. hjorti* | 99.9 | KY973332 | (Gjerde et al. 2017b) | *S. hjorti* | MT737911 |
|  | D25 clone5 | 654 | *S. hjorti* | 99.5 | KF831294 | (Gjerde 2014a) | *S. hjorti* | MT737912 |
| Deer 26 | D26 clone1 | 666 | *S. venatoria*  *S. iberica* | 99.6  99.6 | KY973327  KY973321 | (Gjerde et al. 2017b)  (Gjerde et al. 2017b) | *Sarcocystis* sp. | MW020010 |
|  | D26 clone2 | 654 | *S. hjorti* | 99.9 | GQ250990 | (Dahlgren and Gjerde 2010a) | *S. hjorti* | MW020009 |
|  | D26 clone3 | 666 | *S. venatoria*  *S. iberica* | 99.4  99.4 | KY973327  KY973321 | (Gjerde et al. 2017b)  (Gjerde et al. 2017b) | *Sarcocystis* sp. | MW020011 |
|  | D26 clone4 | 666 | *S. venatoria*  *S. iberica* | 99.7  99.7 | KY973327  KY973321 | (Gjerde et al. 2017b)  (Gjerde et al. 2017b) | *Sarcocystis* sp. | MW020012 |
|  | D26 clone5 | 662 | *S. venatoria*  *S. iberica* | 99.7  99.6 | KY973324  KY973318 | (Gjerde et al. 2017b)  (Gjerde et al. 2017b) | *Sarcocystis* sp. | MW020013 |
| Deer 29 | D29 clone1 | 636 | *S. silva* | 99.7 | KY019065 | (Gjerde et al. 2017a) | *S. silva* | MT737913 |
|  | D29 clone2 | 648 | *S. silva* | 98.3 | KY019059 | (Gjerde et al. 2017a) | *Sarcocystis* sp*.* | MT737914 |
|  | D29 clone3 | 645 | *S. silva* | 98.6 | KY019067 | (Gjerde et al. 2017a) | *Sarcocystis* sp*.* | MT737915 |
|  | D29 clone4 | 637 | *S. silva* | 99.7 | KY019056 | (Gjerde et al. 2017a) | *S. silva* | MT737916 |
|  | D29 clone5 | 664 | *S. venatoria* | 99.1 | KY973325 | (Gjerde et al. 2017b) | *Sarcocystis* sp*.* | MT737917 |
|  |  |  | *S. iberica* | 98.8 | KY973318 | (Gjerde et al. 2017b) |  |  |
| Deer 30 | D30 clone1 | 664 | *S. venatoria* | 99.4 | KY973325 | (Gjerde et al. 2017b) | *Sarcocystis* sp*.* | MT737918 |
|  |  |  | *S. iberica* | 99.1 | KY973323 | (Gjerde et al. 2017b) |  |  |
|  | D30 clone2 | 661 | *S. iberica* | 99.9 | KY973318 | (Gjerde et al. 2017b) | *Sarcocystis* sp*.* | MT737919 |
|  |  |  | *S. venatoria* | 99.7 | KY973324 | (Gjerde et al. 2017b) |  |  |
|  | D30 clone3 | 664 | *S. venatoria* | 99.6 | KY973325 | (Gjerde et al. 2017b) | *Sarcocystis* sp*.* | MT737920 |
|  |  |  | *S. iberica* | 99.3 | KY973318 | (Gjerde et al. 2017b) |  |  |
|  | D30 clone4 | 664 | *S. venatoria* | 99.7 | KY973325 | (Gjerde et al. 2017b) | *Sarcocystis* sp*.* | MT737921 |
|  |  |  | *S. iberica* | 99.4 | KY973318 | (Gjerde et al. 2017b) |  |  |
|  | D30 clone5 | 666 | *S. venatoria* | 99.4 | KY973327 | (Gjerde et al. 2017b) | *Sarcocystis* sp*.* | MT737922 |
|  |  |  | *S. iberica* | 99.4 | KY973321 | (Gjerde et al. 2017b) |  |  |
| Deer 32 | D32 clone1 | 654 | *S. hjorti* | 99.7 | KF831294 | (Gjerde 2014a) | *S. hjorti* | MT737923 |
|  | D32 clone2 | 654 | *S. hjorti* | 99.7 | KF831294 | (Gjerde 2014a) | *S. hjorti* | MT737924 |
|  | D32 clone3 | 654 | *S. hjorti* | 99.7 | KF831294 | (Gjerde 2014a) | *S. hjorti* | MT737925 |
|  | D32 clone5 | 654 | *S. hjorti* | 99.9 | KF831294 | (Gjerde 2014a) | *S. hjorti* | MT737926 |
| Deer 33 | D33 clone1 | 652 | *S. hjorti* | 99.9 | KY973332 | (Gjerde et al. 2017b) | *S. hjorti* | MT737927 |
|  | D33 clone2 | 654 | *S. hjorti* | 99.7 | KF831294 | (Gjerde 2014a) | *S. hjorti* | MT737928 |
|  | D33 clone4 | 654 | *S. hjorti* | 99.7 | KF831294 | (Gjerde 2014a) | *S. hjorti* | MT737928 |
| Deer 34 | D34 clone1 | 652 | *S. hjorti* | 99.1 | KY973332 | (Gjerde et al. 2017b) | *S. hjorti* | MT737929 |
|  | D34 clone2 | 654 | *S. hjorti* | 99.9 | KF831294 | (Gjerde 2014a) | *S. hjorti* | MT737930 |
|  | D34 clone5 | 654 | *S. hjorti* | 99.7 | KF831294 | (Gjerde 2014a) | *S. hjorti* | MT737931 |

Direct sequencing of PCR products from Deer 3, 8, 9 and 26 revealed sequence ambiguities, suggesting mixed infection with different *Sarcocystis* species, but cloning of these products was not attempted. Samples from Deer 27, 28 and 31 (control group) yielded negative PCR results for *Sarcocystis*.

ID: identification; bp: base pairs; in Sequence ID: “PCR” refers to a sequence obtained by direct sequencing and “clone” to a sequence obtained by cloning of a PCR product in a plasmid vector.

**Supplementary Table 5**: Description of *Sarcocystis* spp. 18S rRNA gene sequences amplified from *Sarcocystis* oocysts/sporocysts from faeces and intestinal mucosa of red foxes or faeces of hunting dogs from Grisons, Switzerland, obtained by direct sequencing of the PCR products or after cloning into vector plasmids

| Animal  ID | Sequence ID | Sequence  length (bp) | BLASTn identity | (%) | GenBank®  accession no.  (reference  sequence) | Reference | *Sarcocystis* sp.  (this study) | GenBank®  accession no.  (this study) |
| --- | --- | --- | --- | --- | --- | --- | --- | --- |
| Fox 1 | Fox1 PCR | 623 | *S. tenella* | 100 | KP263759 | (Kolenda et al. 2015) | *S. tenella* | MT737932 |
|  | Fox1 clone1 | 647 | *S. tenella* | 99.7 | MK420019 | (Gjerde et al. 2020) | *S. tenella* | MW020893 |
|  | Fox1 clone2 | 646 | *S. tenella* | 99.7 | KP263759 | (Kolenda et al. 2015) | *S. tenella* | MW020894 |
|  | Fox1 clone3 | 642 | *S. capracanis*  *S. tenella* | 93.8  93.4 | KU820983  MK420019 | (Hu et al. 2016)  (Gjerde et al. 2020) | *Sarcocystis* sp. | MW020895 |
|  | Fox1 clone4 | 647 | *S. tenella* | 99.9 | KP263759 | (Kolenda et al. 2015) | *S. tenella* | MW020896 |
|  | Fox1 clone5 | 647 | *S. tenella* | 99.7 | KP263759 | (Kolenda et al. 2015) | *S. tenella* | MW020897 |
| Fox 10 | Fox10 PCR | 625 | *S. tenella* | 100 | KP263759 | (Kolenda et al. 2015) | *S. tenella* | MT737933 |
|  | Fox10 clone1 | 646 | *S. capracanis*  *S. tenella* | 98.5  98.3 | KU820983  MF039329 | (Hu et al. 2016)  (Hu et al. 2017) | *Sarcocystis* sp. | MW020898 |
|  | Fox10 clone3 | 648 | *S. tenella* | 99.2 | MF039329 | (Hu et al. 2017) | *S. tenella* | MW020899 |
|  | Fox10 clone4 | 647 | *S. tenella* | 100 | KP263759 | (Kolenda et al. 2015) | *S. tenella* | MW020900 |
| Fox 16 | Fox16 PCR | 639 | *S. miescheriana* | 100 | MH404232 | (Gazzonis et al. 2019) | *S. miescheriana* | MT737934 |
|  | Fox16 clone1 | 664 | *S. miescheriana* | 100 | MH404232 | (Gazzonis et al. 2019) | *S. miescheriana* | MW020901 |
|  | Fox16 clone5 | 664 | *S. miescheriana* | 99.9 | MH404232 | (Gazzonis et al. 2019) | *S. miescheriana* | MW020902 |
|  | Fox16 clone7 | 664 | *S. miescheriana* | 99.9 | MH404232 | (Gazzonis et al. 2019) | *S. miescheriana* | MW020903 |
|  | Fox16 clone8 | 664 | *S. miescheriana* | 100 | MH404232 | (Gazzonis et al. 2019) | *S. miescheriana* | MW020901 |
| Fox 46 | Fox46 PCR | 617 | *S. hjorti* | 100 | GQ250990 | (Dahlgren and Gjerde 2010a) | *S. hjorti* | MT737935 |
| Fox 47 | Fox47 PCR | 638 | *S. gracilis* | 100 | MN334289 | (Rudaitytė-Lukošienė et al. 2020) | *S. gracilis* | MT737936 |
|  | Fox47 clone1 | 675 | *S. gracilis* | 99.7 | MN334289 | (Rudaitytė-Lukošienė et al. 2020) | *S. gracilis* | MW020904 |
|  | Fox47 clone2 | 675 | *S. gracilis* | 99.7 | MN334289 | (Rudaitytė-Lukošienė et al. 2020) | *S. gracilis* | MW020905 |
|  | Fox47 clone3 | 675 | *S. gracilis* | 100 | MN334289 | (Rudaitytė-Lukošienė et al. 2020) | *S. gracilis* | MW020906 |
|  | Fox47 clone4 | 675 | *S. gracilis* | 99.7 | MN334289 | (Rudaitytė-Lukošienė et al. 2020) | *S. gracilis* | MW020907 |
|  | Fox47 clone5 | 647 | *S. tenella* | 99.9 | KP263756 | (Kolenda et al. 2015) | *S. tenella* | MW020908 |
| Fox 108 | Fox108 PCR | 624 | *S. tenella* | 100 | KP263759 | (Kolenda et al. 2015) | *S. tenella* | MT737937 |
|  | Fox108 clone1 | 647 | *S. capracanis*  *S. tenella* | 98.9  98.8 | KU820983  MF039329 | (Hu et al. 2016)  (Hu et al. 2017) | *Sarcocystis* sp. | MW020909 |
|  | Fox108 clone2 | 651 | *S. hircicanis*  *S. aretiecanis* | 95.2  94.5 | KU820984  MK420017 | (Hu et al. 2016)  (Gjerde et al. 2020) | *Sarcocystis* sp. | MW020910 |
|  | Fox108 clone3 | 651 | *S. hircicanis*  *S. aretiecanis* | 95.2  94.6 | KU820984  MK420017 | (Hu et al. 2016)  (Gjerde et al. 2020) | *Sarcocystis* sp. | MW020911 |
|  | Fox108 clone4 | 647 | *S. tenella* | 99.9 | MK420019 | (Gjerde et al. 2020) | *S. tenella* | MW020912 |
|  | Fox108 clone5 | 647 | *S. tenella* | 99.7 | MK420019 | (Gjerde et al. 2020) | *S. tenella* | MW020913 |
| Fox 112 | Fox112 PCR | 621 | *S. tenella* | 100 | MK420019 | (Gjerde et al. 2020) | *S. tenella* | MT737938 |
|  | Fox112 clone 4 | 648 | *S. tenella* | 99.4 | MK420019 | (Gjerde et al. 2020) | *S. tenella* | MW020914 |
|  | Fox112 clone 5 | 647 | *S. tenella* | 99.5 | KP263759 | (Kolenda et al. 2015) | *S. tenella* | MW020915 |
|  | Fox112 clone 6 | 647 | *S. capracanis*  *S. tenella* | 98.9  98.6 | KU820983  KP263759 | (Hu et al. 2016)  (Kolenda et al. 2015) | *Sarcocystis* sp. | MW020916 |
|  | Fox112 clone 7 | 648 | *S. tenella* | 99.5 | MF039329 | (Hu et al. 2017) | *S. tenella* | MW020917 |
|  | Fox112 clone 8 | 647 | *S. tenella* | 99.5 | MK420019 | (Gjerde et al. 2020) | *S. tenella* | MW020918 |
| Fox 115 | Fox115 PCR | 637 | *S. gracilis* | 100 | MN334289 | (Rudaitytė-Lukošienė et al. 2020) | *S. gracilis* | MT737939 |
| Fox 120 | Fox120 PCR | 621 | *S. tenella* | 100 | KP263759 | (Kolenda et al. 2015) | *S. tenella* | MT737940 |
|  | Fox120 clone1 | 647 | *S. tenella* | 99.7 | MF039329 | (Hu et al. 2017) | *S. tenella* | MW020919 |
|  | Fox120 clone7 | 647 | *S. tenella* | 99.2 | KP263759 | (Kolenda et al. 2015) | *S. tenella* | MW020920 |
|  | Fox120 clone8 | 645 | *S. tenella*  *S. capracanis* | 98.9  98.8 | KP263759  KU820982 | (Kolenda et al. 2015)  (Hu et al. 2016) | *Sarcocystis* sp. | MW020921 |
|  | Fox120 clone9 | 647 | *S. tenella* | 99.7 | KP263756 | (Kolenda et al. 2015) | *S. tenella* | MW020922 |
|  | Fox120 clone10 | 647 | *S. capracanis*  *S. tenella* | 98.9  98.6 | KU820983  KP263756 | (Hu et al. 2016)  (Kolenda et al. 2015) | *Sarcocystis* sp. | MW020923 |
| Fox 125 | Fox125 PCR | 624 | *S. tenella* | 100 | KP263759 | (Kolenda et al. 2015) | *S. tenella* | MT737941 |
|  | Fox125 clone1 | 647 | *S. tenella* | 99.9 | MF039329 | (Hu et al. 2017) | *S. tenella* | MW021141 |
|  | Fox125 clone4 | 647 | *S. tenella* | 99.9 | MK420019 | (Gjerde et al. 2020) | *S. tenella* | MW021142 |
|  | Fox125 clone5 | 647 | *S. tenella* | 100 | MK420019 | (Gjerde et al. 2020) | *S. tenella* | MW021143 |
|  | Fox125 clone6 | 647 | *S. tenella* | 100 | MK420019 | (Gjerde et al. 2020) | *S. tenella* | MW021143 |
|  | Fox125 clone7 | 647 | *S. tenella* | 99.1 | MK420019 | (Gjerde et al. 2020) | *S. tenella* | MW021144 |
| Fox 126 | Fox126 PCR | 622 | *S. tenella* | 100 | KP236759 | (Kolenda et al. 2015) | *S. tenella* | MT737942 |
|  | Fox126 clone5 | 647 | *S. tenella* | 99.2 | MF039329 | (Hu et al. 2017) | *S. tenella* | MW020924 |
|  | Fox126 clone6 | 642 | *S. capracanis*  *S. tenella* | 98.5  98.3 | KU820983  MF039329 | (Hu et al. 2016)  (Hu et al. 2017) | *Sarcocystis* sp. | MW020925 |
|  | Fox126 clone8 | 647 | *S. capracanis*  *S. tenella* | 93.6  93.2 | KU820983  MK420019 | (Hu et al. 2016)  (Gjerde et al. 2020) | *Sarcocystis* sp. | MW020926 |
|  | Fox126 clone9 | 647 | *S. tenella* | 99.4 | KP263758 | (Kolenda et al. 2015) | *S. tenella* | MW020927 |
| Fox 6 | Fox6 clone1 | 647 | *S. capracanis* | 96.6 | KU820983 | (Hu et al. 2016) | *Sarcocystis* sp. | MT737943 |
|  |  |  | *S. tenella* | 98.5 | MF039329 | (Hu et al. 2017) |  |  |
|  | Fox6 clone2 | 652 | *S. hjorti* | 99.7 | KY973332 | (Gjerde et al. 2017b) | *S. hjorti* | MT737944 |
|  | Fox6 clone3 | 652 | *S. hjorti* | 100 | KY973332 | (Gjerde et al. 2017b) | *S. hjorti* | MT737945 |
|  | Fox6 clone4 | 654 | *S. hjorti* | 99.5 | KF831294 | (Gjerde 2014a) | *S. hjo*r*ti* | MT737946 |
|  | Fox6 clone5 | 647 | *S. tenella* | 99.9 | MK420019 | (Gjerde et al. 2020) | *Sarcocystis* sp. | MT737947 |
|  |  |  | *S. capracanis* | 99.2 | L76472 | (Jeffries et al. 1997) |  |  |
| Fox 15 | Fox15 clone1 | 645 | *S. capracanis* | 99.5 | KU820982 | (Hu et al. 2016) | *Sarcocystis* sp. | MT737948 |
|  |  |  | *S. tenella* | 99.1 | KP263759 | (Kolenda et al. 2015) |  |  |
|  | Fox15 clone2 | 649 | *S. capracanis* | 98.9 | KU820983 | (Hu et al. 2016) | *Sarcocystis* sp. | MT737949 |
|  |  |  | *S. tenella* | 97.4 | MF039329 | (Hu et al. 2017) |  |  |
|  | Fox15 clone3 | 649 | *S. capracanis* | 98.7 | KU820983 | (Hu et al. 2016) | *Sarcocystis* sp. | MT737950 |
|  |  |  | *S. tenella* | 97.1 | MK420018 | (Gjerde et al. 2020) |  |  |
|  | Fox15 clone4 | 645 | *S. capracanis* | 99.7 | KU820982 | (Hu et al. 2016) | *Sarcocystis* sp. | MT737951 |
|  |  |  | *S. tenella* | 99.2 | KP263759 | (Kolenda et al. 2015) |  |  |
|  | Fox15 clone5 | 642 | *S. hircicanis* | 96.3 | KU820984 | (Hu et al. 2016) | *Sarcocystis* sp. | MT737952 |
|  |  |  | *S. aretiecanis* | 95.8 | MK420017 | (Gjerde et al. 2020) |  |  |
| Dog 5 | Dog5 clone1 | 663 | *S. capreolicanis* | 99.5 | KY019029 | (Gjerde et al. 2017a) | *S. capreolicanis* | MT737953 |
|  | Dog5 clone2 | 675 | *S. gracilis* | 99.4 | MN334289 | (Rudaitytė-Lukošienė et al. 2020) | *S. gracilis* | MT737954 |
|  | Dog5 clone3 | 675 | *S. gracilis* | 99.1 | MN334289 | (Rudaitytė-Lukošienė et al. 2020) | *S. gracilis* | MT737955 |
|  | Dog5 clone5 | 663 | *S. capreolicanis* | 99.4 | MN334253 | (Rudaitytė-Lukošienė et al. 2020) | *S. capreolicanis* | MT737956 |
| Dog 6 | Dog6 clone1 | 656 | *S. linearis* | 99 | MN334294 | (Rudaitytė-Lukošienė et al. 2020) | *Sarcocystis* sp. | MT737957 |
|  |  |  | *S. taeniata* | 98.9 | KU753890 | (Prakas et al. 2016) |  |  |
|  | Dog6 clone3 | 656 | *S. linearis* | 98.9 | MN334294 | (Rudaitytė-Lukošienė et al. 2020) | *Sarcocystis* sp. | MT737958 |
|  |  |  | *S. taeniata* | 98.7 | KU753890 | (Prakas et al. 2016) |  |  |
|  | Dog6 clone9 | 675 | *S. gracilis* | 99.8 | MN334289 | (Rudaitytė-Lukošienė et al. 2020) | *S. gracilis* | MT737959 |

Samples from Foxes 2, 12, 33 and 98 yielded negative PCR results for *Sarcocystis*.

ID: identification; bp: base pairs; in Sequence ID: “PCR” refers to a sequence obtained by direct sequencing and “clone” to a sequence obtained by cloning of a PCR product in a plasmid vector.

**References**

Dahlgren, S.S., Gjerde, B., 2010a. Molecular characterization of five *Sarcocystis* species in red deer (*Cervus elaphus*), including *Sarcocystis hjorti* n. sp., reveals that these species are not intermediate host specific. Parasitology, 137, 815–840.

Gazzonis, A.L., Gjerde, B., Villa, L., Minazzi, S., Zanzani, S. A., Riccaboni, P., Sironi G., Manfredi M.T. 2019. Prevalence and molecular characterisation of *Sarcocystis miescheriana* and *Sarcocystis* *suihominis* in wild boars (*Sus scrofa*) in Italy. Parasitol. Res. 118, 1271–1287.

Gjerde, B., 2014a. Morphological and molecular characteristics of four *Sarcocystis* spp. in Canadian moose (*Alces alces*), including *Sarcocystis taeniata* n. sp. Parasitol. Res. 113, 1591–1604.

Gjerde, B., Giacomelli, S., Bianchi, A., Bertoletti, I., Mondani, H., Gibelli, L.R., 2017a. Morphological and molecular characterization of four *Sarcocystis* spp., including *Sarcocystis linearis* n. sp., from roe deer (*Capreolus capreolus*) in Italy. Parasitol. Res. 116, 1317–1338.

Gjerde, B., de La Fuente, C., Alunda, J.M., Luzón, M., 2020. Molecular characterisation of five *Sarcocystis* species in domestic sheep (*Ovis aries*) from Spain. Parasitol. Res. 119, 215–231.

Gjerde, B., Luzón, M., Alunda, J.M., de La Fuente, C.,2017b. Morphological and molecular characteristics of six *Sarcocystis* spp. from red deer (*Cervus elaphus*) in Spain, including *Sarcocystis cervicanis* and three new species. Parasitol. Res. 116, 2795–2811.

Hu, J.J., Huang, S., Wen, T., Esch, G.W., Liang, Y., Li, H.L., 2017. *Sarcocystis* spp. chez les moutons domestiques à Kunming, en Chine. Prévalence, morphologie et caractéristiques moléculaires. Parasite (Paris, France) 24, 30.

Hu, J.J., Liu, T.T., Liu, Q., Esch, G.W., Chen, J.Q., Huang, S., Wen, T., 2016. Prevalence, morphology, and molecular characteristics of *Sarcocystis* spp. in domestic goats (*Capra hircus*) from Kunming, China. Parasitol. Res. 115, 3973–3981.

Irie, T., Ichii, O., Nakamura, T., Ikeda, T., Ito, T., Yamazaki, A., Takai S., Yagi, K., 2019. Molecular characterization of three *Sarcocystis* spp. from wild sika deer (*Cervus nippon yesoensis*) in Hokkaido, Japan. Vet. Parasitol. Region. Stud. Rep. 18, 100327.

Irie, T., Ikeda, T., Nakamura, T., Ichii, O., Yamada, N., Ito, T., Yamazaki, A., Takai S., Yagi, K., 2017. First molecular detection of *Sarcocystis ovalis* in the intestinal mucosa of a Japanese jungle crow (*Corvus macrorhynchos*) in Hokkaido, Japan. Vet. Parasitol. Region. Stud. Rep. 10, 54–57.

Jeffries, A.C., Schnitzler, B., Heydorn, A.O., Johnson, A.M., Tenter, A.M., 1997. Identification of synapomorphic characters in the genus *Sarcocystis* based on 18S rDNA sequence comparison. J. Euk. Microbiol. 44, 388–392.

Kolenda, R., Schierack, P., Zieba, F., Zwijacz-Kozica, T., Bednarski, M., 2015. First molecular characterization of *Sarcocystis tenella* in Tatra chamois (*Rupicapra rupicapra tatrica*) in Poland. Parasitol. Res. 114, 3885–3892.

Prakas, P., Butkauskas, D., Rudaitytė, E., Kutkienė, L., Sruoga, A., Pūraitė, I., 2016. Morphological and molecular characterization of *Sarcocystis taeniata* and *Sarcocystis pilosa* n. sp. from the sika deer (*Cervus nippon*) in Lithuania. Parasitol. Res. 115, 3021–3032.

Reissig, E.C., Moré, G., Massone, A., Uzal, F.A. (2016): Sarcocystosis in wild red deer (*Cervus elaphus*) in Patagonia, Argentina. *Parasitol. Res.* 115, 1773–1778.

Rudaitytė-Lukošienė, E., Delgado de Las Cuevas, G.E., Prakas, P., Calero-Bernal, R., Martínez-González, M., Strazdaitė-Žielienė, Ž., Servienė, E., Habela, M. A., Butkauskas, D., 2020. *Sarcocystis* spp. diversity in the roe deer (*Capreolus capreolus*) from Lithuania and Spain. Parasitol. Res.119:1363-1370.
